# Gene–Diet Interaction Analysis in UK Biobank Identified Genetic Loci That Modify the Association Between Fish Oil Supplementation and the Incidence of Dementia

**DOI:** 10.1016/j.cdnut.2025.107524

**Published:** 2025-08-05

**Authors:** Yueqi Lu, Huifang Xu, Yitang Sun, Susan Adanna Ihejirika, Charleston WK Chiang, Burcu F Darst, Suhang Song, Ye Shen, Kaixiong Ye

**Affiliations:** 1 Department of Genetics, Franklin College of Arts and Sciences, University of Georgia, Athens, Georgia, United States; 2 Institute of Bioinformatics, University of Georgia, Athens, Georgia, United States; 3 Center for Genetic Epidemiology, Department of Population and Public Health Sciences, University of Southern California, Los Angeles, California, United States; 4 Public Health Sciences, Fred Hutchinson Cancer Center, Seattle, Washington, United States; 5 Department of Epidemiology, University of Washington, Seattle, Washington, United States; 6 Department of Health Policy and Management, College of Public Health, University of Georgia, Athens, Georgia, United States; 7 Department of Epidemiology and Biostatistics, College of Public Health, University of Georgia, Athens, Georgia, United States

**Keywords:** time-to-event GWAS, gene-by-environment interactions, fish oil supplementation, dementia, prospective cohort study

## Abstract

**Background:**

Dementia is a common disease influenced by both genetic and environmental factors. *APOE* ε4 is well-known to increase risk of dementia, and it has been shown to attenuate the protective association of fish oil supplements (FOS) and the incidence of dementia.

**Objectives:**

To identify additional genetic factors with modifying effects, we performed a genome-wide scan.

**Methods:**

We performed genome-wide association studies (GWAS) of incident all-cause dementia, Alzheimer's disease, and vascular dementia in 357,631 participants from UK Biobank and the FOS subgroups. Single-nucleotide polymorphisms (SNPs) suggestively associated with dementia (*P* < 1 × 10^−5^) were then evaluated for their interactions with fish oil status in Cox regression models. Furthermore, we conducted gene set enrichment analysis to identify the relevant cell types for these interaction signals.

**Results:**

Time-to-event GWAS identified 6, 5, and 2 genome-wide significant loci (*P* < 5 × 10^−8^) for the incidence of all-cause dementia, Alzheimer's disease, and vascular dementia, respectively. Most of them overlapped with previously known GWAS loci for Alzheimer's disease and related dementia. A total of 178 suggestive GWAS loci (*P* < 1 × 10^−5^) were passed onto interaction analysis, and 43 of them were found to significantly modify the association between FOS and dementia incidence (*P* < 2.8 × 10^−4^ with Bonferroni correction). One locus overlapped with a known Alzheimer's disease GWAS locus (*EED*/*PICALM*) and 2 with GWAS loci for circulating ω-3 fatty acids (*SRSF4* and *PSMG1*). Candidate interacting genes exhibited cell-type–specific expression in the nervous system.

**Conclusions:**

In total, 43 genetic loci modify the association between FOS and dementia. These findings indicate a need for genome-informed personalized nutrition of FOS for the purpose of dementia prevention.

## Introduction

Dementia is a significant global health issue exacerbated by population aging and growth [1]. This syndrome, characterized by the loss of cognitive functioning, is influenced by both genetic and environmental factors [[2], [3], [4]]. Nutrition and dietary components are considered modifiable risk factors for dementia [2]. Observational studies reported that both dietary intakes and circulating concentrations of ω-3 (n–3) fatty acids were associated with lower risk of cognitive decline [5]. Habitual intake of fish oil supplements (FOS), a rich source of long-chain ω-3 fatty acids, was found to be inversely associated with risk of all-cause dementia and several subtypes [6]. The impacts of genetic factors have been examined by multiple genome-wide association studies (GWAS) [4,7,8]. A meta-study in 2023 revealed >75 loci associated with incident Alzheimer's disease and related dementias (ADRD) [4]. One notable gene is *APOE*, whose risk allele *APOE* ε4 has been linked to vascular dysfunction, amyloid-β pathology, neurodegeneration, and ultimately, dementia [[9], [10], [11], [12], [13]].

Gene–environment (G × E) interaction studies on human health are gaining attention. These studies focus on how specific genetic and environmental factors synergistically increase disease risk, providing new insights into the etiology of complex diseases and promoting the development of preventative strategies and therapies [14]. Recent studies showed that *APOE* ε4 modifies the protective associations of FOS with incident all-cause dementia and vascular dementia, suggesting that only noncarriers of *APOE* ε4 may benefit from FOS [15,16]. Besides *APOE*, the potential modifying effects of other genetic factors remain unknown.

In this study, we sought to identify additional genetic variants that modify the association of FOS with all-cause dementia and its two main subtypes, Alzheimer's disease and vascular dementia. Time-to-event models used throughout the analysis allowed the detection of genetic variants associated with the onset of diseases [17]. We adopted a two-step approach by first identifying suggestive loci associated with dementia phenotypes in the overall sample and the two FOS subgroups. Then, time-to-event interaction analysis was performed to identify significant interaction loci with Bonferroni correction for multiple testing. Finally, we assessed the expression signatures of candidate genes at these interaction loci and identified their biologically relevant cell types.

## Methods

### Study design and participants

UK Biobank is a large-scale prospective study that recruited ∼500,000 participants aged 40–69 years who lived in the UK between 2006 and 2010 [18]. Phenotypic and genotypic data were collected from questionnaires, interviews, physical measures, and biological samples at baseline. Follow-up data on health-related outcomes were accessed mainly through linkages to their records in available national databases. We included participants of genetic European ancestry, who completed the touchscreen questionnaire regarding FOS status, did not have a diagnosis of dementia at baseline, and did not withdraw from the study by 25 April, 2023. The ancestry background was obtained from the Pan-UK Biobank Project by projecting the UK Biobank participants onto the principal components (PCs) inferred from the reference panels of the Human Genome Diversity Panel and 1000 Genomes Project [19,20]. The UK Biobank received ethical approval from the research ethics committee (reference ID: 11/ NW/0382). Written informed consent was obtained from all participants. This study was conducted using the UK Biobank Resource (application number 48818).

### Outcomes

The dementia outcomes provided by UK Biobank were algorithmically defined using information from self-reported verbal interviews, linked hospital admissions, and death registries [15,16,21]. Our study considered incident all-cause dementia, Alzheimer's disease, and vascular dementia as the longitudinal outcomes. Incidence was defined as the first diagnosed date following the participant’s visit to the assessment center at baseline. The end of follow-up for each participant was recorded as the earliest of the following dates: dementia occurrence, death, or the end of follow-up records (13 December, 2022).

### Exposure

The touchscreen questionnaire completed at the assessment center provided information on FOS status. Participants were asked, “Do you regularly take any of the following?” Participants who selected the option “fish oil (including cod liver oil)” were considered fish oil users, and those who only selected other supplementation or “none of the above” were considered nonfish oil users. We confirmed that fish oil supplementation status is positively associated with the absolute concentration and relative percentage of ω-3 fatty acids in plasma, as reported in previous studies [16,22].

To evaluate the robustness of this questionnaire, we performed a sensitivity analysis on FOS status based on the 24-h dietary recall questionnaire. Participants were invited five times to repeatedly fill out this online questionnaire and were asked, “Did you have any vitamin or mineral supplements yesterday?” We divided all individuals into a 24-h recall subgroup and a touchscreen-only subgroup and performed time-to-event interaction analysis in the two subgroups separately. We also performed interaction analyses using oily fish intake and circulating ω-3 concentrations in place of FOS for sensitivity analyses. Oily fish intake was collected from the touchscreen questionnaire under the question “How often do you eat oily fish? (e.g., sardines, salmon, mackerel, and herring).” The following options were provided for participants to select from the following: never, less than once a week, once a week, 2–4 times a week, 5–6 times a week, and once or more daily. We categorized the responses of never and less than once a week into a low intake group, and the rest into a high intake group. The circulating ω-3 concentration was measured by nuclear magnetic resonance technique in ∼280,000 plasma samples collected at baseline. It was treated as a continuous variable in our sensitivity interaction analyses.

### APOE ε4 dosage

*APOE* ε4 dosage was known to modify the relationship between FOS and dementia onset [15]. The *APOE* genotype was defined by two single-nucleotide polymorphisms (SNPs), rs7412 and rs429358. The dosage of *APOE* ε4 was divided into 0 (ε1/ε1, ε1/ε2, ε2/ε2, ε2/ε3, ε3/ε3), 1 (ε1/ε4, ε3/ε4) and 2 (ε4/ε4), respectively. Because the determination of ε1/ε3 and ε2/ε4 from the genotype data was ambiguous, and ε2/ε4 may lead to a conflated effect, we did not include them in the interaction analysis.

### Genotype data processing

We used the imputed genotype dataset released by UK Biobank in 2018 [23]. About 92 million autosomal variants were imputed with the Haplotype Reference Consortium panel and the UK10K as references. Participants were removed if they had mismatches between self-reported sex and genetically inferred sex, exhibited sex chromosome aneuploidy, or were identified as outliers for heterozygosity or missing rate. We also excluded participants who had excessive third-degree relatives and randomly removed one individual among pairs of related individuals closer than the third-degree relationship.

For marker-based quality control, we first filtered out low-quality imputed variants with an info score of <0.3 and retained only biallelic variants. SNPs with a minor allele frequency (MAF) of <0.1%, a missing calling rate of >5%, and a Hardy-Weinberg equilibrium *P* value of <10^−8^ were also removed. Finally, we ensured that the missing call rate per individual was <2% after these processes. Genotype data was all processed by PLINK, version 1.9 or version 2.0 [24].

### Candidate SNP selection

We performed time-to-event GWAS by a scalable and accurate method, saddlepoint approximation implementation based on the Cox proportional hazards regression model, for all-cause dementia, Alzheimer's disease, and vascular dementia [17]. This model substantially enhances computational efficiency while effectively controlling type I error rates at the genome-wide significance level across MAFs. Age, sex, and the top 10 genetic PCs were included as covariates. Independent loci of genome-wide significant signals (*P* < 5 × 10^−8^) for each outcome were defined based on clumping (*P* < 5 × 10^−8^ and *r*^2^ < 0.1 within a 250-kb physical distance) and then merging if two signal clusters are within 250 kb of each other. The novelty of these loci was defined with comparison with previous GWAS of ADRD [4].

Because we are looking for genetic factors that modify the association between FOS and dementia outcomes, the associations of these genetic factors with dementia outcomes are dependent on the FOS status and may be masked or weakened in the GWAS in the entire data set. Therefore, we also performed time-to-event GWAS of each outcome in the two subgroups of fish oil users and nonusers, separately. Candidate variants for subsequent interaction analysis were selected using a lenient threshold of *P* < 1 × 10^−5^ from GWAS in the entire data set or the two subgroups.

### Time-to-event G × E interaction analysis

Effects of interactions between candidate SNPs and FOS on incident dementia were evaluated using the Cox proportional hazards models. In model 1, the same covariates used in the time-to-event GWAS, including age, sex, top 10 genetic PCs, along with FOS and the corresponding SNP, were included for the interaction analysis. Interaction signals were identified with a Bonferroni-corrected threshold based on the number of independent candidate loci. These loci were obtained from the clumped regions of suggestive signals (*r*^2^ < 0.1; *P* < 1 × 10^−5^ within a 250-kb physical distance) with ±250-kb extension. The *P* threshold of 1 × 10^−5^ is commonly used in genome-wide studies to define suggestive signals [[25], [26], [27]]. The interaction analyses with *APOE* ε4 dosage were performed as positive controls.

Additionally, considering the potential confounding effects of socioeconomic status, lifestyle, dietary patterns, and related medical history, models 2–4 adjusted for additional factors were included as sensitivity analyses. Education (high/low), Townsend deprivation index (TDI), body mass index (BMI), smoking (never/previous/current), alcohol intake (nondrinker/low to moderate drinker/heavy drinker), and physical activity were included in model 2 on the basis of model 1. Model 3 further included 8 dietary patterns: oily fish intake (<1 times per week/1 times per week/≥2 times per week), fruit intake (<2 servings per day/2–3.9 servings per day/≥4 servings per day), vegetable intake (<2 servings per day/2-3.9 servings per day/≥4 servings per day), processed meat intake (<2 times per week/≥2 times per week), red meat intake (<2 times per week/≥2 times per week), vitamin supplementation (yes/no), mineral supplementation (yes/no), and glucosamine supplementation (yes/no) [16]. Self-reported medical histories of five conditions, including hypertension (yes/no), cardiovascular disease (yes/no), high cholesterol (yes/no), diabetes (yes/no), and depression (yes/no), were additionally included in model 4. Self-reported medical histories were collected from verbal interviews at the time of recruitment. Specifically, cardiovascular diseases included angina, heart attack or myocardial infarction, stroke, subarachnoid hemorrhage, brain hemorrhage, and ischemic stroke. Among these covariates, TDI, BMI, and physical activity are continuous variables, and others were treated as unordered categorical variables in models.

### Gene set enrichment analysis

Genome-wide significant interaction signals were annotated by the Variant Effect Predictor toolset on the Ensembl platform [28]. Each candidate variant was assigned location information for the genes, transcripts, and protein sequences that it might affect. Then, gene set enrichment analysis was conducted on the Web-based Cell-type–Specific Enrichment Analysis of Genes (WebCSEA) website for the list of candidate protein-coding genes included in the WebCSEA background gene list, to identify the cell type–specific expression signatures of these interaction signals [29].

### Statistical analysis

Descriptive and inferential analyses were performed using R version 3.4.1 [30]. Imputation of missing covariate data was conducted by the *mice* package using predictive mean matching, logistic regression, and polytomous logistic regression methods for continuous variables, binary variables, and unordered multiple categorical variables, respectively [31].

## Results

**FIGURE 1:**
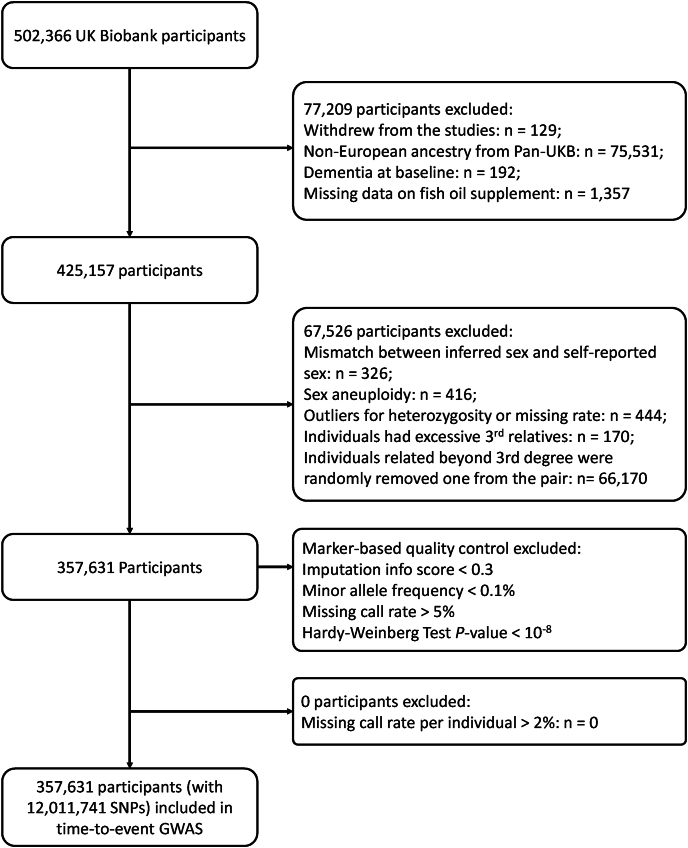
Flow chart of participant inclusion and exclusion. GWAS, genome-wide association study; SNP, single nucleotide polymorphism.

As shown in Figure 1, 425,157 participants of genetic European ancestry from the UK Biobank completed the touchscreen questionnaire on FOS and did not have a diagnosis of dementia at recruitment. After quality control with genotype data and removing related individuals, 67,526 participants were excluded. Finally, 357,631 participants with a mean age of 56.81 ± 8.01 years and a median follow-up period of 13.8 years were included in our study. Around 31.7% of participants reported consuming FOS, as documented in the touchscreen questionnaire at baseline (Supplemental Tables 1 and 2). Older individuals and women appeared more likely to take FOS. Compared with the non-fish oil users, those taking FOS had a lower education level and higher prevalence of hypertension and high cholesterol.

### Associations between fish oil supplementation and incident dementias

We evaluated the associations between FOS and incident all-cause dementia and its subtypes using Cox proportional hazards models over a median follow-up period of 13.8 years. Regular intake of FOS demonstrated protective associations with incident all-cause dementia [hazard ratio (HR): 0.92; 95% confidence interval (CI): 0.87, 0.96] and vascular dementia (HR: 0.80; 95% CI: 0.72, 0.89) after adjustment for age and sex (Supplemental Table 3). In model 4, the model with the most covariates, a consistent relationship was shown in all-cause dementia (HR: 0.94; 95% CI: 0.89, 0.99), although the association between FOS and vascular dementia was no longer significant after controlling for dietary patterns and medical histories (*P* = 0.141). FOS showed no significant association with the onset of Alzheimer's disease across all models.

### Time-to-event GWAS on dementia

**FIGURE 2:**
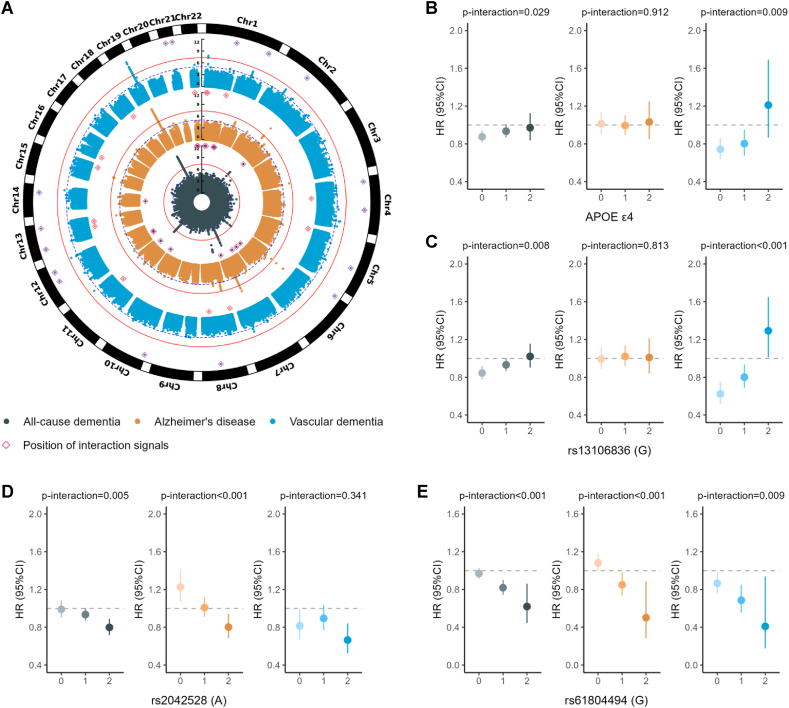
GWAS and G × FOS interaction analysis of dementia. (A) Manhattan plots of time-to-event GWAS for all-cause dementia (dark cyan), Alzheimer's disease (orange), and vascular dementia (blue). Variants with −log10(*P*) above 11 were not shown. Thresholds of genome-wide significance (*P* < 5 × 10^−8^) and suggestive significance (*P* < 1 × 10^−5^) were indicated by solid red lines and dashed blue lines, respectively. Positions of interaction signals were labeled on the top of Manhattan plots. Associations between FOS and incident dementia in genotype subgroups of (B) *APOE* ε4, (C) rs13106836, (D) rs2042528, and (E) rs61804494. The gray dashed lines represented null effects. The values of *P*-interaction were evaluated for the interaction terms SNP × FOS (yes/no) by Cox regression models adjusted by age, sex, and top 10 genetic PCs. CHR, chromosome; HR, hazard ratio; *P*-interaction, *P* value of the interaction terms.

We first performed time-to-event GWAS on three dementia outcomes separately, to identify disease-related loci and select candidate variants for further interaction analysis. In the entire data set of 357,631 participants and at the genome-wide significance (*P* < 5 × 10^−8^), we identified 6 loci for all-cause dementia, 5 for Alzheimer's disease, and 2 for vascular dementia (Figure 2A; Supplemental Table 4). *APOE* was the locus shared among these three dementia outcomes. Bridging integrator 1 (*BIN1*), triggering receptor expressed on myeloid cells 2 (*TREM2*), and zinc finger CW-type (*ZCWPW1*)/PWWP domain containing 1 (*NYAP1*) were identified in both all-cause dementia and Alzheimer's disease. Protein tyrosine kinase 2β (*PTK2B*)/clusterin (*CLU*) and membrane-spanning 4A (*MS4A*) were significantly associated with the onset of all-cause dementia, whereas aph-1 homolog B (*APH1B*) was specifically linked to Alzheimer's disease. A novel locus for vascular dementia was discovered on chromosome 1, with castor zinc finger 1 (*CASZ1*) being the closest gene to the top signal.

Considering that genetic variants with interaction effects have exposure-dependent effects on the onset of dementia, we conducted stratified GWAS for the two subgroups of fish oil users and nonusers. A total of 1,819 suggestive common SNPs (*P* < 1 × 10^−5^; MAF > 0.01) in 178 independent loci were then selected for interaction analysis (Supplemental Figures 1–3).

### Time-to-event gene × FOS interaction analysis

**FIGURE 3:**
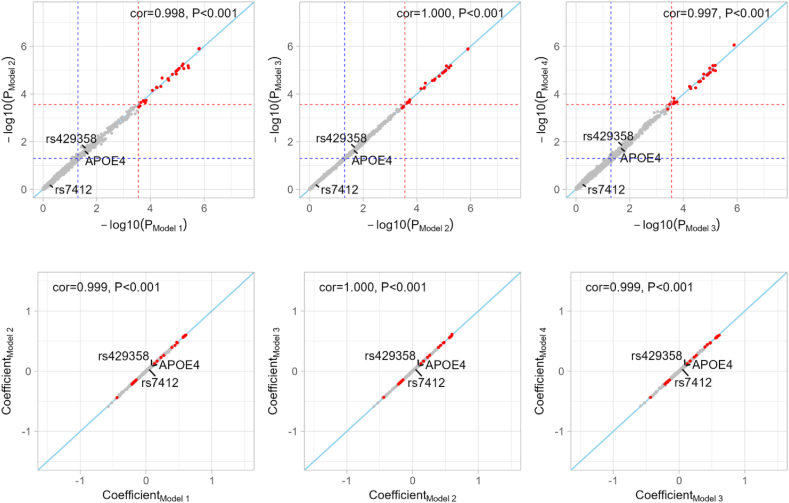
Comparison of results from interaction analysis of candidate SNPs with FOS in all-cause dementia using models 1–4. Model 1 adjusted for basic covariates, including age, sex, top 10 genetic principal components, along with fish oil supplementation and the corresponding SNP. Model 2 further adjusted for education, Townsend deprivation index, BMI, smoking, alcohol intake, and physical activity. Model 3 additionally included 8 dietary patterns: oily fish intake, fruit intake, vegetable intake, processed meat intake, red meat intake, vitamin supplementation, mineral supplementation, and glucosamine supplementation. Model 4 included 5 self-reported medical histories, hypertension, cardiovascular disease (CVD), high cholesterol, diabetes, and depression. The SNPs that significantly interacted with FOS status in model 1 were shown as red points. The red dashed line and blue dashed line referred to the Bonferroni correction threshold (*P* = 2.8 × 10^−4^) and nominal significance threshold (*P* = 0.05), respectively. Pearson tests were conducted to assess the correlation of *P* values or β coefficients between models. cor, Pearson correlation coefficient; P, *P* value of Pearson correlation test.

We performed time-to-event gene × FOS interaction analysis with the selected candidate SNPs for all-cause dementia, Alzheimer's disease, and vascular dementia. Our primary interaction analyses used the Cox proportional hazards model, referred to as model 1, which adjusted for age, sex, FOS status, top 10 genetic PCs, and dosage of the corresponding SNP. A total of 81 SNPs in 43 loci were found to have significant interaction signals (2.8 × 10^−4^, or 0.05/178 candidate loci) for one of the three dementia outcomes (Table 1, Figure 2A, Supplemental Tables 5–8). After adjusting for socioeconomic status, lifestyle, dietary patterns, and related medical history, models that included some or all of these additional covariates yielded similarly significant interaction signals (Figure 3, Supplemental Figures 4 and 5). In model 4, with the most covariates, locus 9 for all-cause dementia, locus 38 for Alzheimer's disease, and locus 30 and locus 31 for vascular dementia were no longer significant.

**TABLE 1:** Genetic loci with significant gene×FOS interaction effects on three dementia outcomes.

CHR	No. loci	Top SNP^1^	Closest gene^2^	REF	ALT	*P*-interaction^3^	HR_FOS_ by carrying REF allele(s)^4^			HR_SNP_ by FOS status^4^	
							0	1	2	Fish oil users	Non-fish oil user
All-cause dementia											
1	2	rs75837905	*KAZN*	A	G	3.70 × 10^−5^	0.89 (0.85, 0.94)	1.44 (1.16, 1.80)	—	1.34 (1.14, 1.57)	0.84 (0.72, 0.97)
1	3	rs116264291	*SRSF4*	A	G	1.78 × 10^−4^	0.90 (0.86, 0.95)	1.42 (1.11, 1.83)	—	1.51 (1.27, 1.81)	0.95 (0.80, 1.13)
1	4	rs61804494	*OLFM3*	G	A	1.45 × 10^−4^	0.97 (0.91, 1.03)	0.82 (0.74, 0.90)	0.62 (0.45, 0.86)	0.84 (0.77, 0.91)	1.01 (0.95, 1.07)
1	5	rs12043527	*PRMT6*	G	A	8.37 × 10^−5^	0.87 (0.83, 0.92)	1.10 (0.98, 1.23)	1.20 (0.79, 1.83)	1.15 (1.05, 1.25)	0.91 (0.85, 0.98)
1	6	rs12024264	*RBM15*	C	A	1.52 × 10^−4^	0.88 (0.84, 0.93)	1.29 (1.10, 1.52)	1.43 (0.52, 3.92)	1.32 (1.17, 1.48)	0.89 (0.80, 1.00)
2	9	rs4435418	*MYO3B*	C	T	2.71 × 10^−4^	0.81 (0.73, 0.91)	0.89 (0.83, 0.95)	1.05 (0.96, 1.15)	1.14 (1.08, 1.21)	1.00 (0.96, 1.05)
6	16	rs9505681	*HS3ST5*	C	T	4.10 × 10^−5^	0.90 (0.85, 0.94)	1.61 (1.21, 2.15)	—	1.64 (1.34, 2.01)	0.90 (0.73, 1.10)
6	17	rs963302	*RPS6KA2*	C	G	3.98 × 10^−4^	1.01 (0.94, 1.08)	0.86 (0.79, 0.93)	0.68 (0.57, 0.82)	0.86 (0.81, 0.92)	1.04 (0.99, 1.09)
7	18	rs3114430	*ITGB8*	C	A	2.09 × 10^−5^	1.13 (0.98, 1.30)	0.95 (0.88, 1.03)	0.81 (0.75, 0.88)	0.88 (0.83, 0.93)	1.03 (0.98, 1.08)
7	19	rs12670543	*OR2A14*	C	A	3.82 × 10^−4^	0.86 (0.82, 0.91)	1.17 (1.04, 1.31)	1.23 (0.74, 2.04)	1.22 (1.12, 1.33)	0.93 (0.86, 1.00)
10	24	rs4750683	*PTPRE*	G	C	2.44 × 10^−4^	1.05 (0.87, 1.26)	1.02 (0.94, 1.11)	0.84 (0.79, 0.90)	0.86 (0.80, 0.91)	0.99 (0.95, 1.04)
11	26	rs3018644	*JRKL*	G	A	6.01 × 10^−5^	1.45 (0.96, 2.18)	1.06 (0.95, 1.17)	0.87 (0.82, 0.92)	0.94 (0.87, 1.02)	1.16 (1.09, 1.24)
12	27	rs74523587	*PCED1B*	A	C	1.54 × 10^−4^	0.90 (0.85, 0.94)	1.40 (1.12, 1.76)	—	1.49 (1.26, 1.76)	0.97 (0.83, 1.13)
12	28	rs73119275	*RP11-762I7.5; DNAJC14*	C	T	6.15 × 10^−4^	0.90 (0.85, 0.94)	1.56 (1.22, 2.00)	—	1.52 (1.27, 1.81)	0.86 (0.72, 1.02)
14	35	rs12896185	*OTX2*	A	G	5.71 × 10^−5^	0.85 (0.80, 0.91)	0.99 (0.91, 1.08)	1.23 (1.00, 1.51)	1.15 (1.08, 1.23)	0.97 (0.93, 1.03)
22	42	rs35137695	*PEX26*	C	T	6.68 × 10^−4^	0.90 (0.85, 0.94)	1.59 (1.22, 2.07)	—	1.64 (1.37, 1.96)	0.90 (0.74, 1.08)
22	43	rs144548824	*AP1B1*	G	A	1.83 × 10^−4^	0.94 (0.89, 0.99)	0.59 (0.47, 0.75)	—	0.86 (0.71, 1.05)	1.33 (1.18, 1.51)
Alzheimer's disease											
1	1	rs116501531	*SLC45A1*	A	G	5.30 × 10^−5^	0.98 (0.91, 1.05)	2.18 (1.48, 3.20)	—	1.92 (1.50, 2.46)	0.87 (0.65, 1.16)
1	2	rs75837905	*KAZN*	A	G	2.15 × 10^−5^	0.97 (0.90, 1.04)	1.95 (1.42, 2.66)	—	1.68 (1.36, 2.06)	0.84 (0.66, 1.06)
1	6	rs12024264	*RBM15*	C	A	2.50 × 10^−5^	0.96 (0.89, 1.03)	1.63 (1.28, 2.06)	1.44 (0.31, 6.57)	1.43 (1.21, 1.68)	0.87 (0.73, 1.03)
4	11	rs149325653	*CENPC*	A	G	1.31 × 10^−4^	1.04 (0.97, 1.12)	0.40 (0.24, 0.65)	—	0.65 (0.42, 1.00)	1.70 (1.36, 2.13)
5	13	rs59781823	*IRX1*	G	A	5.87 × 10^−5^	1.14 (1.03, 1.27)	0.96 (0.86, 1.08)	0.68 (0.54, 0.87)	0.94 (0.86, 1.03)	1.17 (1.10, 1.26)
8	20	rs2042528	*CSMD1*	A	T	7.34 × 10^−5^	1.23 (1.07, 1.41)	1.01 (0.91, 1.12)	0.80 (0.68, 0.94)	0.94 (0.87, 1.02)	1.16 (1.09, 1.24)
8	22	rs116871946	*KCNV1*	T	C	2.06 × 10^−4^	1.05 (0.97, 1.13)	0.68 (0.53, 0.87)	0.10 (0.01, 0.85)	0.88 (0.72, 1.08)	1.38 (1.21, 1.59)
11	25	rs78851816	*EED*	G	A	5.75 × 10^−4^	0.94 (0.87, 1.01)	1.49 (1.24, 1.79)	1.50 (0.67, 3.39)	1.42 (1.24, 1.62)	0.93 (0.82, 1.06)
13	32	rs9571707	*PCDH9*	A	G	1.83 × 10^−4^	0.85 (0.77, 0.94)	1.14 (1.02, 1.27)	1.42 (1.11, 1.81)	1.21 (1.12, 1.32)	0.93 (0.86, 1.00)
13	33	rs306675	*GPC6*	A	C	1.95 × 10^−5^	0.82 (0.69, 0.98)	0.94 (0.84, 1.04)	1.25 (1.10, 1.41)	1.22 (1.12, 1.32)	0.96 (0.90, 1.03)
15	37	rs28698386	*MESP2*	T	C	1.20 × 10^−4^	1.30 (1.13, 1.50)	0.95 (0.86, 1.05)	0.88 (0.75, 1.01)	0.83 (0.77, 0.90)	1.02 (0.96, 1.09)
16	38	rs75422462	*ERCC4*	G	A	2.28 × 10^−4^	0.98 (0.91, 1.06)	1.86 (1.30, 2.67)	—	1.78 (1.40, 2.26)	0.91 (0.69, 1.19)
18	39	rs34352315	*CCDC68*	G	A	3.42 × 10^−5^	1.04 (0.97, 1.12)	0.36 (0.22, 0.60)	—	0.63 (0.40, 1.00)	1.88 (1.50, 2.35)
22	43	rs144548824	*AP1B1*	G	A	2.06 × 10^−4^	1.04 (0.97, 1.12)	0.52 (0.36, 0.74)	—	0.69 (0.50, 0.94)	1.37 (1.14, 1.65)
Vascular dementia											
1	5	rs11184799	*PRMT6*	G	A	5.86 × 10^−5^	0.70 (0.62, 0.80)	1.09 (0.89, 1.34)	1.25 (0.63, 2.46)	1.49 (1.27, 1.74)	0.98 (0.86, 1.11)
1	7	rs17163137	*HHIPL2*	T	C	3.82 × 10^−5^	1.04 (0.88, 1.23)	0.72 (0.61, 0.84)	0.57 (0.43, 0.76)	0.77 (0.68, 0.88)	1.07 (0.98, 1.17)
2	8	rs113777826	*AFF3*	C	T	1.20 × 10^−4^	0.77 (0.69, 0.86)	2.16 (1.27, 3.68)	—	2.39 (1.70, 3.37)	0.84 (0.56, 1.27)
4	10	rs148811174	*LDB2*	C	T	6.38 × 10^−8^	0.99 (0.87, 1.12)	0.53 (0.44, 0.65)	0.48 (0.26, 0.87)	0.73 (0.62, 0.87)	1.27 (1.14, 1.41)
4	12	rs13106836	*CAMK2D*	G	A	5.65 × 10^−4^	0.63 (0.52, 0.75)	0.80 (0.69, 0.93)	1.29 (1.01, 1.65)	1.33 (1.18, 1.50)	0.94 (0.85, 1.02)
5	14	rs2089903	*ST8SIA4*	C	A	7.41 × 10^−7^	—	2.56 (1.59, 4.11)	0.75 (0.68, 0.84)	0.46 (0.34, 0.61)	1.52 (1.05, 2.21)
6	15	rs845890	*AL033381.1*	C	A	1.49 × 10^−4^	0.68 (0.59, 0.78)	0.98 (0.82, 1.17)	1.35 (0.85, 2.13)	1.40 (1.22, 1.61)	1.00 (0.89, 1.11)
8	21	rs117216345	*SNX16*	T	A	2.14 × 10^−5^	0.73 (0.65, 0.83)	1.27 (0.97, 1.66)	1.75 (0.70, 4.39)	1.59 (1.32, 1.93)	0.91 (0.76, 1.08)
10	23	rs116876958	*AKR1E2*	C	T	1.65 × 10^−4^	0.76 (0.68, 0.85)	1.63 (1.11, 2.40)	—	2.03 (1.55, 2.65)	0.98 (0.74, 1.29)
12	29	rs1585705	*YEATS4*	A	C	6.05 × 10^−5^	0.53 (0.38, 0.73)	0.72 (0.61, 0.85)	0.97 (0.83, 1.12)	1.13 (0.99, 1.29)	0.81 (0.74, 0.89)
12	30	rs11833702	*HSP90B1*	A	G	1.96 × 10^−4^	0.74 (0.66, 0.83)	1.21 (0.92, 1.58)	4.86 (1.02, 23.11)	1.63 (1.33, 1.99)	0.97 (0.81, 1.16)
13	31	rs3783124	*ATP8A2*	C	T	2.08 × 10^−4^	0.85 (0.76, 0.95)	0.37 (0.23, 0.59)	—	0.67 (0.44, 1.02)	1.61 (1.32, 1.98)
13	34	rs72653992	*CLYBL*	A	G	5.78 × 10^−5^	0.75 (0.67, 0.84)	1.54 (1.09, 2.18)	1.40 (0.27, 7.27)	2.06 (1.62, 2.61)	1.02 (0.80, 1.29)
14	36	rs28444185	*DIO2*	A	T	8.16 × 10^−5^	0.65 (0.56, 0.76)	0.97 (0.82, 1.13)	1.10 (0.78, 1.55)	1.34 (1.18, 1.52)	0.97 (0.88, 1.07)
21	40	rs2823575	*USP25*	G	A	4.40 × 10^−5^	0.70 (0.62, 0.80)	1.24 (1.00, 1.54)	0.92 (0.39, 2.18)	1.47 (1.24, 1.73)	0.93 (0.80, 1.07)
21	41	rs74482819	*PSMG1*	C	T	1.74 × 10^−5^	0.76 (0.68, 0.85)	2.15 (1.34, 3.45)	—	2.18 (1.61, 2.97)	0.76 (0.53, 1.10)

Abbreviations: ALT, alternate allele; CHR, chromosome; FOS, fish oil supplements; HR, hazard ratio; POS, chromosome base position; REF, reference allele; SNP, single-nucleotide polymorphism.

1 The most significant interaction SNPs in loci for each outcome.

2 Closest genes were identified as the protein-coding genes with the shortest physical distance to the top interaction signal at each locus, based on the GRCh37 genome build from the Ensembl database.

3 The significance of the interaction between FOS and dementia outcomes was evaluated using Cox regression models adjusted by age, sex, top 10 genetic principal components, FOS, and corresponding SNPs’ dosage.

4 The associations between FOS and dementia outcomes, SNPs and dementia outcomes were evaluated using Cox regression models adjusted by age, sex, and top 10 genetic principal components within SNP genotype groups and FOS groups, respectively. Subgroups with 5 or fewer incident cases were not considered in the subgroup analysis.

A total of 22 protein-coding genes from these 43 interaction loci were annotated through the Variant Effect Predictor tool. One locus was annotated to the calcium/calmodulin-dependent protein kinase IIδ (*CAMK2D*) gene, which is highly expressed in the heart muscle tissue. The top variant at this locus, rs13106836 (G/A), exhibited a pattern similar to that of *APOE* ε4 (rs13106836 × FOS interaction *P* = 0.008 for all-cause dementia; interaction *P* < 0.001 for vascular dementia) (Figure 2B, C). Among individuals with no or one G allele, taking FOS was associated with lower risks of all-cause dementia (HR: 0.85; 95% CI: 0.78, 0.92, in individuals of AA genotype) and vascular dementia (HR: 0.80; 95% CI: 0.69, 0.93, in individuals of AA genotype; HR: 0.63; 95% CI: 0.52, 0.75, in individuals of AG genotype), whereas a risk-increasing association was observed in those homozygous for the G allele (HR: 1.29; 95% CI: 1.01, 1.65, in individuals of GG genotype). The association between FOS and Alzheimer's disease was not significant in any of the three genotypes. Other interesting interaction patterns were observed. For example, SNP rs2042528 (A/T) in gene *CSMD1* had significant interaction signals for both all-cause dementia (*P* = 0.005) and Alzheimer's disease (*P* < 0.001), and FOS had opposite association directions with Alzheimer's disease in the two homozygous genotype groups (HR: 1.23; 95% CI: 1.07, 1.41, in individuals of TT genotype; HR: 0.80; 95% CI: 0.68, 0.94, in individuals of AA genotype) (Figure 2D). SNP rs61804494 (G/A) around gene *OLFM3* had significant interaction signals for all three dementia outcomes (all *P* < 0.009), and the protective association effects of FOS with the three outcomes increased linearly with each additional copy of G allele (Figure 2E).

We compared the interaction loci with previously reported GWAS loci for ADRD and circulating polyunsaturated fatty acids (PUFAs). One locus on chromosome 11 overlapped with a reported ADRD locus around gene embryonic ectoderm development (*EED*), and 2 loci on chromosomes 1 and 21 overlapped with known GWAS loci for circulating PUFAs, including serine and arginine-rich splicing factor 4 (*SRSF4*) and proteasome assembly chaperone 1 (*PSMG1*) (Supplemental Table 8). Of note, none of the interaction loci overlapped with the genome-wide significant loci in our time-to-event GWAS on dementia.

Although the total number of interaction loci across the three dementia outcomes was 43, the numbers were 17, 14, and 16 for all-cause dementia, Alzheimer's disease, and vascular dementia, respectively (Supplemental Figure 6, Supplemental Table 9). Among them, 15, 12, and 15 loci (88%, 86%, and 94%, respectively, of all interaction loci) reached suggestive significance (*P* < 1 × 10^−5^) in GWAS in only one of the two fish oil subgroups. No loci reached suggestive significance in both subgroups. Interestingly, these interaction loci were more likely to exhibit genetic associations in the FOS subgroup: 13 of the 15 loci (87%, *P* = 0.0074) for all-cause dementia and 12 of the 15 loci for vascular dementia (80%, *P* = 0.035) showing suggestive significance exclusively in the fish oil users but not in the nonusers. Alzheimer's disease did not display such a preference, with 7 of 12 loci (58%) exhibiting suggestive significance only in fish oil users. Additionally, we found that half of the significant interaction loci consistently indicated protective effects of FOS on outcomes. At the remaining loci, the FOS–dementia association showed opposite directions, with inverse association in some genotype subgroups but positive association in others. In summary, FOS–interacting genetic loci for all-cause dementia and vascular dementia show more genotype–phenotype associations under FOS. Patterns of both allele-additive protective effects and allele-dependent inverse effects of FOS on dementias were observed at these interaction loci.

### Sensitivity analyses

We performed a series of sensitivity analyses to evaluate the robustness of the identified interaction signals. Besides performing models with additional covariates that we mentioned earlier, we also compared the interaction signals from model 1 using different exposures, including FOS status defined by 24-h recall questionnaire, oily fish intake, and circulating ω-3 concentrations. There were 20 loci that were replicated with ≥1 alternative exposure (Supplemental Table 10). With the FOS status from the 24-h dietary recall, 3 loci for all-cause dementia, 2 loci for Alzheimer's disease, and 7 loci for vascular dementia were replicated. The numbers of replicated loci were 2, 1, and 2 with oily fish intake, and 3, 3, and 7 with circulating ω-3 concentrations for all-cause dementia, Alzheimer's disease, and vascular dementia, respectively. Among these replicated loci, *EED* locus was replicated with both 24-h recall FOS and ω-3 concentrations in Alzheimer's disease development. Loci *CAMK2D* and ATPase phospholipid transporting 8A2 (*ATP8A2*) were also replicated with these two exposures for vascular dementia, whereas loci iodothyronine deiodinase 2 (*DIO2*) and *PSMG1* were replicated with oily fish intake and ω-3 concentrations. From the comparison of FOS exclusively defined by two questionnaires, interaction signals around peroxisomal biogenesis factor 26 (*PEX26*), *EED*, and *CAMK2D* were replicated in all-cause dementia, Alzheimer's disease, and vascular dementia outcomes, respectively (Supplemental Figure 7).

### Gene set enrichment analysis

**FIGURE 4:**
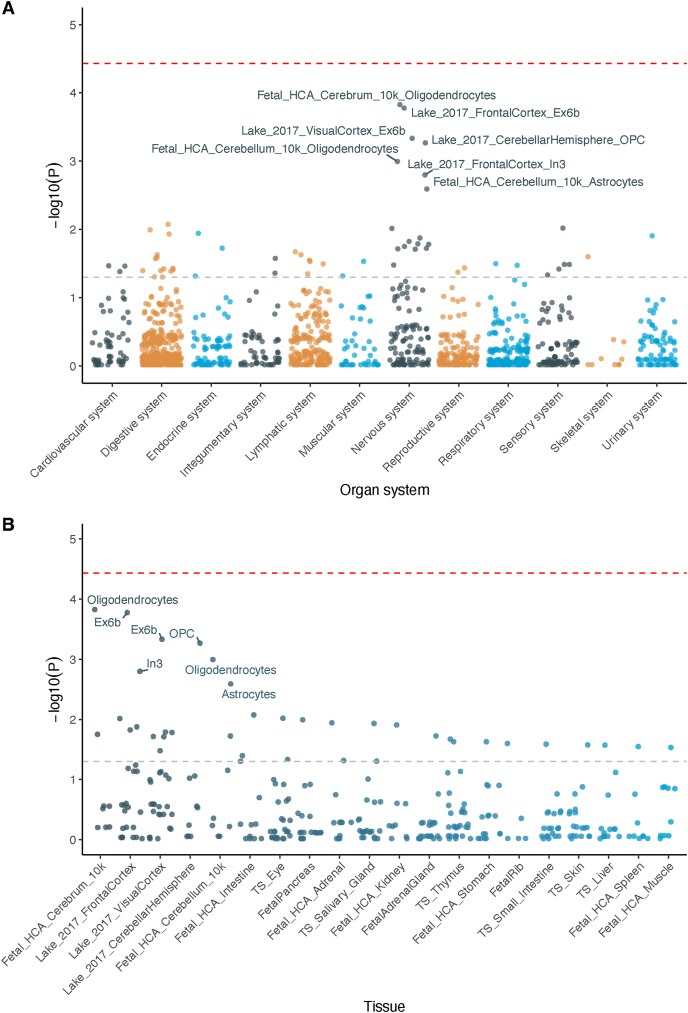
Gene set enrichment analysis of cell-type–specific expression for candidate interacting, protein-coding genes. (A) Enrichment signals stratified by organ systems. (B) Enrichment signals stratified by tissues. Only tissues with the top 20 enrichment signals were shown. Red and gray dashed lines represent thresholds for Bonferroni-corrected significance (0.05/total number of tissue–cell types) and nominal significance (0.05), respectively. Ex6b, layer 6b excitatory neuron subtype; HCA, human cell atlas; In3, layer 3 inhibitory neuron subtype; OPC, oligodendrocyte progenitor cell; *P*, permutated *P* value of gene set enrichment analysis; TS, Tabula Sapiens.

Gene set enrichment analysis was performed on candidate interacting genes to identify their expression signatures across 111 human tissue panels and 1,355 tissue–cell types spanning 12 human organ systems using the WebCSEA website. For the 21 candidate interacting genes that are protein-coding and present in the background gene list of the tool, cell type–specific expression patterns were observed for various cell types of the nervous system (Figure 4, Supplemental Figure 8). The most significant enrichment signal was found in oligodendrocytes in the fetal cerebrum (*P* = 1.49 × 10^−4^). Consistently, oligodendrocytes in the fetal cerebellum (*P* = 1.01 × 10^−3^) and oligodendrocyte progenitor cells (OPCs) in the adult cerebellar hemisphere (*P* = 5.39 × 10^−4^) also had enrichment signals. The second and third most significant enrichment signals were found in the layer 6b excitatory neuron subtype (Ex6b) in the frontal and visual cortex (*P* = 1.67 × 10^−4^ and 4.64 × 10^−4^, respectively). Moreover, cell type–specific expression signals were identified for the layer 3 inhibitory neuron subtype (In3) in the adult frontal cortex (*P* = 1.59 × 10^−3^) and astrocytes in the fetal cerebellum (*P* < 2.56 × 10^−3^). In summary, candidate interacting genes exhibited cell type–specific expression in oligodendrocytes, Ex6b and In3 subtypes of neurons, and in astrocytes.

## Discussion

In this study, we identified 6, 5, and 2 independent genetic loci associated with incident all-cause dementia, Alzheimer's disease, and vascular dementia, respectively. All these loci, except one around *CASZ1*, were reported in the previous ADRD GWAS meta-analysis [4]. Among 178 suggestive GWAS loci, 43 significantly modified the associations between FOS and dementia outcomes. Three loci were previously known to be associated with ADRD or circulating PUFAs [4,[32], [33], [34], [35], [36]]. However, none of these loci overlapped with the time-to-event GWAS loci we found, indicating that the interaction loci may have been missed in typical GWAS. Additionally, candidate protein-coding genes at the interaction loci were enriched for cell type–specific expression for cell types in the nervous system, especially the Ex6b neuron subtype and oligodendrocytes.

*APOE* was the strongest locus associated with all-cause dementia and its two major subtypes in the time-to-event GWAS. It plays a key role in lipid metabolism and homeostasis. One of the major isoforms, *APOE* ε4 is known to be positively associated with amyloid plaques and neurofibrillary tangles formation, contributing to the pathogenesis of Alzheimer's disease [37,38]. Other known Alzheimer's disease–associated genes, *BIN1*, *TREM2*, *ZCWPW1*, and *APH1B*, were also replicated in our time-to-event GWAS [4]. These genes were differentially expressed in specific cell types in the brains of patients with Alzheimer's disease compared with those in healthy individuals [39]. Additionally, we identified a novel locus exclusively associated with vascular dementia. The top SNP within this locus is an intergenic variant likely involved in gene regulation. *CASZ1*, located in this region, plays roles in both neural and cardiovascular development [40]. This gene was reported to be associated with hypertension in both European and Asian populations from previous GWAS [41,42]. It is known that hypertension increases risk of vascular dementia, leading to cognitive decline and cerebrovascular impairment [43,44]. Our GWAS finding of *CASZ1* reveals a shared genetic locus between these two conditions, suggesting a potential target of intervention to manage them simultaneously. Other genes at this unknown locus, such as TAR DNA binding protein (*TARDBP*) and MBL-associated serine protease 2 (*MASP2*), showed significant SNP × SNP interactions with genes involved in τ-related pathologies [45].

We identified 43 interaction loci modifying the associations between FOS and dementia outcomes at statistical significance stronger than that of *APOE* ε4. These findings suggested a need and potential strategies for personalizing fish oil recommendations for high-risk populations. Among the interaction loci, *EED*, *SRSF4*, and *PSMG1*, were reported to be associated with incident ADRD and circulating PUFA traits, including ω-3 PUFA concentration or percentage, and the ω-6:ω-3 ratio. These suggested that the interaction signals may be involved in dementia pathology and PUFA metabolism, although the specific mechanism is still unclear. Notably, none of the interaction loci overlapped with the time-to-event GWAS loci identified in our study. GWAS captures the marginal genetic effects of variants in a sample of participants without explicit consideration of context-dependent genetic effects. Some genetic loci have opposite directions of effects on a phenotype depending on the presence or absence of an environmental factor. This kind of gene–environment interaction results in weak marginal genetic effects, which would not be detected in a typical GWAS. Therefore, directly investigating G × E relationships based solely on known or genome-wide significant GWAS loci may have missed these interaction signals. Instead, we included candidate SNPs from subgroup-specific GWAS to consider the possibility of different or opposite effects of SNPs on outcomes between fish oil users and nonusers, increasing the chance of identifying the interaction signals. None of the interaction loci achieved suggestive significance in both GWAS in the two FOS subgroups. For all-cause dementia and vascular dementia, the majority of the interaction loci only showed GWAS association in the fish oil users, indicating context-specific genetic effects. We proposed that these genotypes or loci may be involved in fatty acid or lipid metabolism pathways. When individuals take FOS, these pathways process the dietary ω-3 fatty acids differently depending on genotype, whereas in the absence of supplementation, no differences are observed across genotype subgroups. We also observed some allele-dependent associations where the FOS effects on dementia were in opposite directions across genotype groups. We suspect that some of these associations may be due to reverse causation, that is, these genotypes could be linked to predementia conditions, such as elevated blood pressure, which motivate individuals to take FOS, resulting in positive associations between FOS and dementia outcomes. Future mechanistic studies of the interaction loci will enhance our understanding of the roles of FOS in the development of dementia. The presence of interaction loci also suggests a need and possibility of improving genetic prediction of dementia risk depending on FOS status.

About half of the 43 FOS-interacting loci were replicated with ≥1 alternative exposure related to dietary or circulating ω-3 fatty acids, demonstrating the robustness of our findings. *CAMK2D*, *ATP8A2*, and *DIO2* loci, along with 2 previously reported GWAS loci, *EED* and *PSMG1*, showed significant interactions with two exposures. *CAMK2D* encodes the δ chain of the CAMKII protein, which is a key mediator for intercellular signaling pathways initiated by calcium signals [46]. This protein is found to regulate lipid metabolism, affecting fatty acid oxidation, whereas also maintaining synaptic plasticity and memory function [[47], [48], [49], [50], [51]]. *ATP8A2* encodes a protein from the P_4_-ATPase family, which is involved in lipid flipping, generating, and maintaining asymmetry in membrane lipids to support various cellular activities [52]. Recently, two mutations on *ATP8A2* were reported to be associated with various neurodevelopmental disorders through protein misfolding [[53], [54], [55]]. *DIO2* encodes deiodinase type II (D2), which catalyzes the conversion of prohormone thyroxine to bioactive 3′,3′,5′-triiodothyronine (T3). These thyroid hormones, especially T3, play roles in hepatic lipid homeostasis, including inducing hepatic lipolysis and regulating fatty acid oxidation [56]. Although *DIO2* polymorphisms and reduced circulating thyroid hormones showed association with Alzheimer's disease and Schizophrenia [[57], [58], [59]]. The biological relevance of these candidate genes further supports the plausibility of our interaction findings.

Candidate protein-coding genes affected by interaction signals were enriched for cell type–specific expression in oligodendrocytes, OPCs, and the Ex6b neuron subtype in adult brain tissues. OPCs can divide and regenerate oligodendrocytes for myelin repair and myelination. This process slows down with aging and is associated with reduced myelination in Alzheimer's disease, contributing to increased cognitive decline [[60], [61], [62]]. The balance of excitatory and inhibitory inputs onto cortical neurons is strictly regulated by homeostatic mechanisms [63]. A recent study revealed that the positive feedback loop between hyperexcitability and amyloidosis may lead to neuronal dysfunction and neurodegeneration [64]. Additionally, ω-3 fatty acids have also been reported to attenuate loss of myelin basic protein and preserve nerve fibers to protect against myelin sheath damage [65]. Further, ω-3 supplementation was found to counteract the decline of subunits on the brain signaling receptors to promote synaptic plasticity [66,67]. Therefore, we speculated that genetic factors and FOS may play synergistic roles in myelin maintenance and synaptic homeostasis. Additionally, we found that half of the interaction signals were mainly located in intronic or intergenic regions, suggesting gene regulation as their possible molecular mechanism.

In this study, we mainly focused on all-cause dementia and its two subtypes, Alzheimer's disease and vascular dementia, in the European population due to the limitations of sample and case sizes. Further studies on different populations and dementia subtypes, such as frontotemporal dementia, are needed to explore similarities and differences in dementia pathogenesis and across ancestry groups. Moreover, we applied a two-step approach to select a subset of SNPs for further time-to-event interaction analysis, reducing the computational cost of genome-wide interaction analysis of longitudinal outcomes. However, this approach was limited to categorical exposure and had the possibility of missing potential interaction signals during the candidate loci selection process. More effective algorithms need to be developed for broader and faster screening. Additionally, the sensitivity analyses revealed a partial lack of replication as certain interaction loci were not reproduced with other related exposures. The differences in the sample sizes across exposures may diminish the replication rate because subtle interaction effects demand large sample sizes for sufficient statistical power. Moreover, the self-reported exposures did not capture dosage details and may be prone to inaccuracies, resulting in false-negative results. We included a more reliable indicator, circulating ω-3 concentrations, as an alternative exposure for validation, but the differences between dietary intake and circulating biomarkers may still result in distinct interaction signals driven by different pathways. We removed related individuals to avoid bias in estimating allele frequencies and thus false discoveries. However, this removed valuable samples and reduced the sample size. More advanced linear mixed methods, which properly control for genetic relatedness, will increase statistical power in future studies. Lastly, it is known that participants in the UK Biobank are healthier than the general population [68]. Our findings may not be generalizable and require future verification in independent cohorts.

In summary, we identified 43 genomic loci that modify the association between FOS and dementia. Gene set enrichment analysis showed that protein-coding genes around the interaction signals demonstrated cell type–specific expression signatures in several cell types in brain tissues. This study revealed interaction relationships between genetic factors and FOS on the development of dementia. It provides new insights into the roles of PUFAs in the pathogenesis of dementia and calls for genome-informed personalized dietary recommendations for dementia prevention.

## Author contributions

The authors’ responsibilities were as follows – KY: designed and conceived the study; HX, YSun: data acquisition; YL: data extraction, cleaning, and analysis; KY, YL: interpreted data and drafted the manuscript; BFD, SS, YShen, CWKC, HX, SAI: provided critical review of the manuscript; and all authors: have read and approved the final manuscript.

## Data availability

The data sets analyzed during this study are available from the UK Biobank through an application process (www.ukbiobank.ac.uk/).

## Funding

Research reported in this publication was supported by the National Institute of General Medical Sciences of the National Institute of Health under the award number R35GM143060 (to KY). The content is solely the responsibility of the authors and does not necessarily represent the official views of the National Institutes of Health.

## Conflict of interest

The authors report no conflict of interest.

## References

[1] GBD (2022). 2019 Dementia Forecasting Collaborators, Estimation of the global prevalence of dementia in 2019 and forecasted prevalence in 2050: an analysis for the Global Burden of Disease Study 2019. Lancet Public Health.

[2] Livingston G., Huntley J., Sommerlad A., Ames D., Ballard C., Banerjee S. (2020). Dementia prevention, intervention, and care: 2020 report of the Lancet Commission. Lancet.

[3] Bellenguez C., Grenier-Boley B., Lambert J.C. (2020). Genetics of Alzheimer’s disease: where we are, and where we are going. Curr. Opin. Neurobiol..

[4] Bellenguez C., Küçükali F., Jansen I.E., Kleineidam L., Moreno-Grau S., Amin N. (2022). New insights into the genetic etiology of Alzheimer’s disease and related dementias. Nat. Genet..

[5] Wei B.Z., Li L., Dong C.W., Tan C.C., Xu W., Alzheimer’s Disease Neuroimaging Initiative (2023). The relationship of omega-3 fatty acids with dementia and cognitive decline: evidence from prospective cohort studies of supplementation, dietary intake, and blood markers. Am. J. Clin. Nutr..

[6] Huang Y., Deng Y., Zhang P., Lin J., Guo D., Yang L. (2022). Associations of fish oil supplementation with incident dementia: evidence from the UK Biobank cohort study. Front. Neurosci..

[7] Andrews S.J., Fulton-Howard B., Goate A. (2020). Interpretation of risk loci from genome-wide association studies of Alzheimer’s disease. Lancet Neurol.

[8] (2024). Mega Vascular Cognitive Impairment and Dementia (MEGAVCID) consortium, A genome-wide association meta-analysis of all-cause and vascular dementia. Alzheimers Dement.

[9] Montagne A., Nikolakopoulou A.M., Huuskonen M.T., Sagare A.P., Lawson E.J., Lazic D. (2021). APOE4 accelerates advanced-stage vascular and neurodegenerative disorder in old Alzheimer’s mice via cyclophilin A independently of amyloid-β. Nat. Aging..

[10] Fortea J., Pegueroles J., Alcolea D., Belbin O., Dols-Icardo O., Vaqué-Alcázar L. (2024). APOE4 homozygozity represents a distinct genetic form of Alzheimer’s disease. Nat. Med..

[11] McCorkindale A.N., Mundell H.D., Guennewig B., Sutherland G.T. (2022). Vascular dysfunction is central to Alzheimer’s disease pathogenesis in APOE e4 carriers. Int. J. Mol. Sci..

[12] Huynh T.V., Davis A.A., Ulrich J.D., Holtzman D.M. (2017). Apolipoprotein E and Alzheimer’s disease: the influence of apolipoprotein E on amyloid-beta and other amyloidogenic proteins. J. Lipid Res..

[13] Shi Y., Yamada K., Liddelow S.A., Smith S.T., Zhao L., Luo W. (2017). ApoE4 markedly exacerbates tau-mediated neurodegeneration in a mouse model of tauopathy. Nature.

[14] Virolainen S.J., VonHandorf A., Viel K.C., Weirauch M.T., Kottyan L.C. (2023). Gene-environment interactions and their impact on human health. Genes Immun..

[15] Ma H., Zhou T., Li X., Heianza Y., Qi L. (2022). Use of fish oil supplements is differently related to incidence of all-cause and vascular dementia among people with the distinct APOE epsilon4 dosage. Clin. Nutr..

[16] He Y., Huang S.Y., Wang H.F., Zhang W., Deng Y.T., Zhang Y.R. (2023). Circulating polyunsaturated fatty acids, fish oil supplementation, and risk of incident dementia: a prospective cohort study of 440,750 participants. Geroscience.

[17] Bi W., Fritsche L.G., Mukherjee B., Kim S., Lee S. (2020). A fast and accurate method for genome-wide time-to-event data analysis and its application to UK Biobank. Am. J. Hum. Genet..

[18] Sudlow C., Gallacher J., Allen N., Beral V., Burton P., Danesh J. (2015). UK Biobank: an open access resource for identifying the causes of a wide range of complex diseases of middle and old age. PLoS Med.

[19] Karczewski K.J., Gupta R., Kanai M., Lu W., Tsuo K., Wang Y. (2024). Pan-UK Biobank GWAS improves discovery, analysis of genetic architecture, and resolution into ancestry-enriched effects. medRxiv.

[20] Donoghue L.J., Benner C., Chang D., Irudayanathan F.J., Pendergrass R.K., Yaspan B.L. (2025). Integration of biobank-scale genetics and plasma proteomics reveals evidence for causal processes in asthma risk and heterogeneity. Cell Genom.

[21] Wilkinson T., Schnier C., Bush K., Rannikmäe K., Henshall D.E., Lerpiniere C. (2019). Identifying dementia outcomes in UK Biobank: a validation study of primary care, hospital admissions and mortality data. Eur. J. Epidemiol..

[22] Aldoori J., Zulyniak M.A., Toogood G.J., Hull M.A. (2024). Fish oil supplement use modifies the relationship between dietary oily fish intake and plasma n-3 PUFA levels: an analysis of the UK Biobank. Br. J. Nutr..

[23] Bycroft C., Freeman C., Petkova D., Band G., Elliott L.T., Sharp K. (2017). Genome-wide genetic data on ∼500,000 UK Biobank participants. bioRxiv.

[24] Chang C.C., Chow C.C., Tellier L.C., Vattikuti S., Purcell S.M., Lee J.J. (2015). Second-generation PLINK: rising to the challenge of larger and richer datasets. Gigascience.

[25] Bonfante B., Faux P., Navarro N., Mendoza-Revilla J., Dubied M., Montillot C. (2021). A GWAS in Latin Americans identifies novel face shape loci, implicating VPS13B and a Denisovan introgressed region in facial variation. Sci. Adv..

[26] Savage J.E., Jansen P.R., Stringer S., Watanabe K., Bryois J., de Leeuw C.A. (2018). Genome-wide association meta-analysis in 269,867 individuals identifies new genetic and functional links to intelligence. Nat. Genet..

[27] Takahashi Y., Yamazaki K., Kamatani Y., Kubo M., Matsuda K., Asai S. (2021). A genome-wide association study identifies a novel candidate locus at the DLGAP1 gene with susceptibility to resistant hypertension in the Japanese population. Sci. Rep..

[28] McLaren W., Gil L., Hunt S.E., Riat H.S., Ritchie G.R., Thormann A. (2016). The Ensembl variant effect predictor. Genome Biol.

[29] Dai Y., Hu R., Liu A., Cho K.S., Manuel A.M., Li X. (2022). WebCSEA: web-based cell-type-specific enrichment analysis of genes. Nucleic Acids Res.

[30] R Core Team, R: (2023).

[31] van Buuren S., Groothuis-Oudshoorn K. (2011). mice: Multivariate Imputation by Chained Equations in R. J. Stat. Softw..

[32] Karjalainen M.K., Karthikeyan S., Oliver-Williams C., Sliz E., Allara E., Fung W.T. (2024). Genome-wide characterization of circulating metabolic biomarkers. Nature.

[33] Richardson T.G., Leyden G.M., Wang Q., Bell J.A., Elsworth B., Davey Smith G. (2022). Characterising metabolomic signatures of lipid-modifying therapies through drug target Mendelian randomisation. PLoS Biol.

[34] Francis M., Sun Y., Xu H., Brenna J.T., Ye K. (2022). Fifty-one novel and replicated GWAS loci for polyunsaturated and monounsaturated fatty acids in 124,024 Europeans. medRxiv.

[35] Borges M.C., Haycock P.C., Zheng J., Hemani G., Holmes M.V., Davey Smith G. (2022). Role of circulating polyunsaturated fatty acids on cardiovascular diseases risk: analysis using Mendelian randomization and fatty acid genetic association data from over 114,000 UK Biobank participants. BMC Med.

[36] Davyson E., Shen X., Gadd D.A., Bernabeu E., Hillary R.F., McCartney D.L. (2023). Metabolomic investigation of major depressive disorder identifies a potentially causal association with polyunsaturated fatty acids. Biol. Psychiatry..

[37] Huang Y., Mahley R.W. (2014). Apolipoprotein E: structure and function in lipid metabolism, neurobiology, and Alzheimer’s diseases. Neurobiol. Dis. 72 Pt.

[38] Blumenfeld J., Yip O., Kim M.J., Huang Y. (2024). Cell type-specific roles of APOE4 in Alzheimer’s disease. Nat. Rev. Neurosci..

[39] Marques-Coelho D., Iohan L., Melo de Farias A.R., Flaig A., Lambert J.C., Brainbank Neuro–CEB Neuropathology Network (2021). Differential transcript usage unravels gene expression alterations in Alzheimer’s disease human brains. NPJ Aging Mech. Dis..

[40] Liu T., Li T., Ke S. (2023). Role of the CASZ1 transcription factor in tissue development and disease. Eur. J. Med. Res..

[41] Irvin M.R., Sitlani C.M., Floyd J.S., Psaty B.M., Bis J.C., Wiggins K.L. (2019). Genome-wide association study of apparent treatment-resistant hypertension in the CHARGE Consortium: the CHARGE Pharmacogenetics Working Group. Am. J. Hypertens..

[42] Kato N., Takeuchi F., Tabara Y., Kelly T.N., Go M.J., Sim X. (2011). Meta-analysis of genome-wide association studies identifies common variants associated with blood pressure variation in east Asians. Nat. Genet..

[43] Emdin C.A., Rothwell P.M., Salimi-Khorshidi G., Kiran A., Conrad N., Callender T. (2016). Blood pressure and risk of vascular dementia: evidence from a primary care registry and a cohort study of transient ischemic attack and stroke. Stroke.

[44] Pacholko A., Iadecola C. (2024). Hypertension, neurodegeneration, and cognitive decline. Hypertension.

[45] Wang H., Yang J., Schneider J.A., De Jager P.L., Bennett D.A., Zhang H.Y. (2020). Genome-wide interaction analysis of pathological hallmarks in Alzheimer’s disease. Neurobiol. Aging..

[46] Berridge M.J., Lipp P., Bootman M.D. (2000). The versatility and universality of calcium signalling. Nat. Rev. Mol. Cell Biol..

[47] Yong J., Song J. (2024). CaMKII activity and metabolic imbalance-related neurological diseases: focus on vascular dysfunction, synaptic plasticity, amyloid beta accumulation, and lipid metabolism. Biomed. Pharmacother..

[48] Zhang J., Xu S., Fang H., Wu D., Ouyang C., Shi Y. (2025). CAMKIIdelta reinforces lipid metabolism and promotes the development of B cell lymphoma. Adv. Sci (Weinh)..

[49] Joseph J.S., Anand K., Malindisa S.T., Fagbohun O.F. (2021). Role of CaMKII in the regulation of fatty acids and lipid metabolism. Diabetes Metab. Syndr..

[50] Ghosh A., Giese K.P. (2015). Calcium/calmodulin-dependent kinase II and Alzheimer’s disease. Mol. Brain..

[51] Takemoto-Kimura S., Suzuki K., Horigane S.I., Kamijo S., Inoue M., Sakamoto M. (2017). Calmodulin kinases: essential regulators in health and disease. J. Neurochem..

[52] Coleman J.A., Quazi F., Molday R.S. (2013). Mammalian P4-ATPases and ABC transporters and their role in phospholipid transport. Biochim. Biophys. Acta..

[53] Matsell E., Andersen J.P., Molday R.S. (2024). Functional and in silico analysis of ATP8A2 and other P4-ATPase variants associated with human genetic diseases. Dis. Model Mech..

[54] Sebastian T.T., Baldridge R.D., Xu P., Graham T.R. (2012). Phospholipid flippases: building asymmetric membranes and transport vesicles. Biochim. Biophys. Acta..

[55] van der Mark V.A., Elferink R.P., Paulusma C.C. (2013). P4 ATPases: flippases in health and disease. Int. J. Mol. Sci..

[56] Marino L., Kim A., Ni B., Celi F.S. (2025). Thyroid hormone action and liver disease, a complex interplay. Hepatology.

[57] Quinlan P., Horvath A., Wallin A., Svensson J. (2019). Low serum concentration of free triiodothyronine (FT3) is associated with increased risk of Alzheimer’s disease. Psychoneuroendocrinology.

[58] McAninch E.A., Rajan K.B., Evans D.A., Jo S., Chaker L., Peeters R.P. (2018). A common DIO_2_ polymorphism and Alzheimer disease dementia in African and European Americans. J. Clin. Endocrinol. Metab..

[59] Akan G., Adolf I.C., Colak A., Acar S., Oncu F., Yesilbursa D. (2025). Thyroid hormone dynamics and DIO_2_ variants in schizophrenia: exploring genetic links to neuroendocrine imbalance. J. Cell. Mol. Med..

[60] Vanzulli I., Papanikolaou M., De-La-Rocha I.C., Pieropan F., Rivera A.D., Gomez-Nicola D. (2020). Disruption of oligodendrocyte progenitor cells is an early sign of pathology in the triple transgenic mouse model of Alzheimer’s disease. Neurobiol. Aging..

[61] Zou P., Wu C., Liu T.C., Duan R., Yang L. (2023). Oligodendrocyte progenitor cells in Alzheimer’s disease: from physiology to pathology. Transl. Neurodegener..

[62] Gong Z., Bilgel M., Kiely M., Triebswetter C., Ferrucci L., Resnick S.M. (2023). Lower myelin content is associated with more rapid cognitive decline among cognitively unimpaired individuals. Alzheimers Dement.

[63] Xue M., Atallah B.V., Scanziani M. (2014). Equalizing excitation-inhibition ratios across visual cortical neurons. Nature.

[64] Scaduto P., Lauterborn J.C., Cox C.D., Fracassi A., Zeppillo T., Gutierrez B.A. (2023). Functional excitatory to inhibitory synaptic imbalance and loss of cognitive performance in people with Alzheimer’s disease neuropathologic change. Acta Neuropathol.

[65] Pu H., Guo Y., Zhang W., Huang L., Wang G., Liou A.K. (2013). Omega-3 polyunsaturated fatty acid supplementation improves neurologic recovery and attenuates white matter injury after experimental traumatic brain injury. J. Cereb. Blood Flow Metab..

[66] Zhou L., Xiong J.Y., Chai Y.Q., Huang L., Tang Z.Y., Zhang X.F. (2022). Possible antidepressant mechanisms of omega-3 polyunsaturated fatty acids acting on the central nervous system. Front. Psychiatry..

[67] Dyall S.C., Michael G.J., Whelpton R., Scott A.G., Michael-Titus A.T. (2007). Dietary enrichment with omega-3 polyunsaturated fatty acids reverses age-related decreases in the GluR2 and NR2B glutamate receptor subunits in rat forebrain. Neurobiol. Aging..

[68] Fry A., Littlejohns T.J., Sudlow C., Doherty N., Adamska L., Sprosen T. (2017). Comparison of sociodemographic and health-related characteristics of UK Biobank participants with those of the general population. Am. J. Epidemiol..

