# Supplementary material for: Gene–Diet Interaction Analysis in UK Biobank Identified Genetic Loci That Modify the Association Between Fish Oil Supplementation and the Incidence of Dementia
Source: Curr Dev Nutr. 2025 Aug 5;9(9):107524. doi: 10.1016/j.cdnut.2025.107524 (PMC12423404; doi:10.1016/j.cdnut.2025.107524)
Supplement: Multimedia component 1 [file mmc1.pdf]

Gene-diet interaction analysis in UK Biobank identified genetic loci that modify the association between fish oil supplementation and the incidence of dementia

Yueqi Lu; Huifang Xu; Yitang Sun; Susan Adanna Ihejirika; Charleston W. K. Chiang; Burcu F. Darst; Suhang Song; Ye Shen; Kaixiong Ye

Supplementary Materials

Table of Content

Table S1. Characteristics of the participants by FOS status. 3

Table S2. Characteristics of the participants by FOS status after imputation. 5

Table S3. Associations between FOS and incident all-cause dementia, Alzheimer's disease, and vascular dementia. 6

Table S4. New and known GWAS loci related to all-cause dementia, Alzheimer's disease, and vascular dementia. 7

Table S5. Associations between FOS and the onset of all-cause dementia in genotype subgroups of SNPs in 43 loci and *APOE* ε4. 8

Table S6. Associations between FOS and the onset of Alzheimer's disease in genotype subgroups of SNPs in 43 loci and *APOE* ε4. 13

Table S7. Associations between FOS and the onset of vascular dementia in genotype subgroups of SNPs in 43 loci and *APOE* ε4. 18

Table S8. 43 Loci interacting with FOS in the development of dementia compared to known GWAS loci. 23

Table S9. Association between interaction loci and dementia outcomes in the whole dataset and FOS subgroups. 25

Table S10. Replicated loci from interaction analyses of FOS status from 24-hour recall questionnaire, oily fish intake, and circulating omega-3 levels. 27

Figure S1. Manhattan plots of GWAS of all-cause dementia by FOS status. 29

Figure S2. Manhattan plots of GWAS of Alzheimer's disease by FOS status. 30

Figure S3. Manhattan plots of GWAS of vascular dementia by FOS status. 31

Figure S4. Comparison of results from interaction analysis of candidate SNPs with FOS in Alzheimer's disease using Model 1-4. 32

Figure S5. Comparison of results from interaction analysis of candidate SNPs with FOS in vascular dementia using Model 1-4. 33

Figure S6. Comparison of the associations between interaction loci and dementia outcomes stratified by FOS status. 34

Figure S7. Interaction analysis of candidate SNPs with FOS status from the touchscreen questionnaire and 24-hour recall questionnaire (model 1). 35

Figure S8. Gene-set enrichment analysis of cell-type specific expression for candidate interacting, protein-coding genes, stratified by adult and fetal tissues. 36

Reference 37

Table S1. Characteristics of the participants by FOS status.

	Fish oil user (N = 113,267)		Non fish oil user (N = 244,364)		*p*-value
	Missing value, n (%)	Values	Missing value, n (%)	Values	
All-cause dementia, n (%)		2,613 (2.31)		4,345 (1.78)	<0.001
Alzheimer's disease, n (%)		1,250 (1.10)		1,848 (0.76)	<0.001
Vascular dementia, n (%)		542 (0.48)		1,015 (0.42)	0.008
Frontotemporal dementia, n (%)		57 (0.05)		162 (0.07)	0.085
Age, years	0	58.94±7.29	0	55.82±8.14	<0.001
Female, n (%)	0	63,514 (56.07)	0	129,047 (52.81)	<0.001
Body mass index, kg/m^2	312 (0.3)	27.15±4.51	843 (0.3)	27.49±4.87	<0.001
Socioeconomic factors					
Education, n (%)	1,023 (0.9)		1,970 (0.8)		<0.001
High		51,008 (45.44)		119,909 (49.47)	
Low		61,236 (54.56)		122,485 (50.53)	
Townsend deprivation index	97 (0.1)	-2.48 [-3.81, -0.24]	346 (0.1)	-2.24 [-3.70, 0.32]	<0.001
Lifestyle factors					
Smoking, n (%)	415 (0.4)		812 (0.3)		
Never		60,396 (53.52)		133,167 (54.68)	<0.001
Previous		43,634 (38.66)		82,629 (33.93)	<0.001
Current		8,822 (7.82)		27,756 (11.40)	<0.001
Alcohol intake, n (%)	74 (0.1)		164 (0.1)		<0.001
Non-drinker		18,537 (16.38)		42,392 (17.36)	
Low to moderate drinker		69,729 (61.60)		149,769 (61.33)	
Heavy drinker		24,927 (22.02)		52,039 (21.31)	
Physical activity, MET-min/week	24,872 (22)	2,013.00 [956.00, 3,879.00]	53,395 (21.9)	1,706.00 [773.00, 3,432.00]	<0.001
Dietary patterns					
Oily fish intake, n (%)	374 (0.3)		1,205 (0.5)		<0.001
<1 times/week		40,594 (35.96)		116,441 (47.89)	
1 times/week		47,079 (41.70)		88,617 (36.44)	
≥2 times/week		25,220 (22.34)		38,101 (15.67)	
Fruits, n (%)	1,233 (1.1)		2,744 (1.1)		<0.001
<2 servings/day		31,481 (28.10)		93,942 (38.88)	
2-3.9 servings/day		57,811 (51.60)		112,069 (46.38)	
≥4 servings/day		22,742 (20.30)		35,609 (14.74)	
Vegetables, n (%)	1,671 (1.5)		4,204 (1.7)		<0.001
<2 servings/day		73,797 (66.13)		170,422 (70.96)	
2-3.9 servings/day		34,469 (30.89)		63,356 (26.38)	
≥4 servings/day		3,330 (2.98)		6,382 (2.66)	
Processed meat, n (%)	117 (0.1)		373 (0.2)		
<2 times/week		80,457 (71.11)		163,525 (67.02)	<0.001
≥2 times/week		32,693 (28.89)		80,466 (32.98)	<0.001
Red meats, n (%)	693 (0.6)		1,758 (0.7)		

<2 times/week		75,033 (66.65)		159,875 (65.90)	<0.001
≥2 times/week		37,541 (33.35)		82,731 (34.10)	<0.001
Vitamins supplementation, n (%)	609 (0.5)	63,050 (55.97)	492 (0.2)	48,461 (19.87)	<0.001
Minerals supplementation, n (%)	0	23,432 (20.69)	0	19,609 (8.02)	<0.001
Glucosamine supplementation, n (%)	0	44,179 (39.00)	0	25,895 (10.60)	<0.001
Self-reported medical histories					
Hypertension, n (%)	0	31,669 (27.96)	0	63,439 (25.96)	<0.001
Cardiovascular disease, n (%)	0	6,357 (5.61)	0	13,630 (5.58)	0.680
High cholesterol, n (%)	0	15,564 (13.74)	0	27,981 (11.45)	<0.001
Diabetes, n (%)	0	4,905 (4.33)	0	11,818 (4.84)	<0.001
Depression, n (%)	0	6,164 (5.44)	0	14,524 (5.94)	<0.001

Normally distributed continuous variables were presented as mean ± standard deviation, non-normally distributed continuous variables were presented as median [interquartile range], and categorical variables were presented as frequency (percentage). And the difference between fish oil intake status groups was estimated by the Wilcoxon rank sum test or Chi-squared Test. Abbreviation: MET, metabolic equivalent task.

Table S2. Characteristics of the participants by FOS status after imputation.

	Fish oil user (N = 113,267)	Non-fish oil user (N = 244,364)	*p*-value
Age, years	58.94±7.29	55.82±8.14	<0.001
Female, n (%)	63,514 (56.07)	129,047 (52.81)	<0.001
Education, n (%)			<0.001
High	51,443 (45.42)	120,710 (49.40)	
Low	61,824 (54.58)	123,654 (50.60)	
Townsend deprivation index	-2.48 [-3.81, -0.24]	-2.24 [-3.70, 0.32]	<0.001
Body mass index, kg/m^2	27.15±4.52	27.49±4.87	<0.001
Smoking, n (%)			
Never	60,608 (53.51)	133,589 (54.67)	<0.001
Previous	43,798 (38.67)	82,937 (33.94)	<0.001
Current	8,861 (7.82)	27,838 (11.39)	<0.001
Alcohol intake, n (%)			<0.001
Non-drinker	18,550 (16.38)	42,424 (17.36)	
Low to moderate drinker	69,776 (61.60)	149,861 (61.33)	
Heavy drinker	24,941 (22.02)	52,079 (21.31)	
Physical activity, MET-min/week	1,986.00 [933.00, 3,828.00]	1,710.00 [777.00, 3,450.00]	<0.001
Oily fish intake, n (%)			<0.001
<1 times/week	40,768 (35.99)	117,057 (47.90)	
1 times/week	47,213 (41.68)	89,037 (36.44)	
≥2 times/week	25,286 (22.32)	38,270 (15.66)	
Fruits, n (%)			<0.001
<2 servings/day	31,871 (28.14)	95,029 (38.89)	
2-3.9 servings/day	58,413 (51.57)	113,320 (46.37)	
≥4 servings/day	22,983 (20.29)	36,015 (14.74)	
Vegetables, n (%)			<0.001
<2 servings/day	74,999 (66.21)	173,494 (71.00)	
2-3.9 servings/day	34,899 (30.81)	64,374 (26.34)	
≥4 servings/day	3,369 (2.97)	6,496 (2.66)	
Processed meat, n (%)			
<2 times/week	80,535 (71.10)	163,755 (67.01)	<0.001
≥2 times/week	32,732 (28.90)	80,609 (32.99)	<0.001
Red meats, n (%)			
<2 times/week	75,492 (66.65)	161,011 (65.89)	<0.001
≥2 times/week	37,775 (33.35)	83,353 (34.11)	<0.001
Vitamins supplementation, n (%)	63,290 (55.88)	48,630 (19.90)	<0.001
Minerals supplementation, n (%)	23,432 (20.69)	19,609 (8.02)	<0.001
Glucosamine supplementation, n (%)	44,179 (39.00)	25,895 (10.60)	<0.001

Normally distributed continuous variables were presented as mean ± standard deviation, non-normally distributed continuous variables were presented as median [interquartile range], and categorical variables were presented as frequency (percentage). And the differences between fish oil intake status groups were estimated by Wilcoxon rank sum test or Chi-squared Test.
Abbreviation: MET, metabolic equivalent task.

Table S3. Associations between FOS and incident all-cause dementia, Alzheimer's disease, and vascular dementia.

	**HR (95%CI)**	***p*-value**
All-cause dementia		
Model 1	0.92 (0.87,0.96)	<0.001
Model 2	0.95 (0.90,1.00)	0.034
Model 3	0.93 (0.88,0.98)	0.009
Model 4	0.94 (0.89,0.99)	0.029
Alzheimer's disease		
Model 1	1.01 (0.94,1.08)	0.863
Model 2	1.03 (0.95,1.10)	0.491
Model 3	1.00 (0.92,1.08)	0.916
Model 4	1.00 (0.93,1.09)	0.928
Vascular dementia		
Model 1	0.80 (0.72,0.89)	<0.001
Model 2	0.85 (0.77,0.95)	0.003
Model 3	0.89 (0.80,1.00)	0.058
Model 4	0.92 (0.82,1.03)	0.141

The associations between fish oil supplementation and dementia onsets were estimated using Cox regression models. Model 1 was adjusted for basic covariates, age, sex, top 10 genetic principal components, along with fish oil supplementation and the corresponding SNP. Model 2 was further adjusted for education, Townsend deprivation index (TDI), body mass index (BMI), smoking, alcohol intake, and physical activity. Model 3 additionally included 8 dietary patterns: oily fish intake, fruit intake, vegetable intake, processed meat intake, red meat intake, vitamin supplementation, mineral supplementation, and Glucosamine supplementation. Model 4 included 5 self-reported medical histories, hypertension, cardiovascular disease (CVD), high cholesterol, diabetes, and depression.

Abbreviations: HR, hazard ratio; CI, confidence interval.

Table S4. New and known GWAS loci related to all-cause dementia, Alzheimer's disease, and vascular dementia.

Outcome	No.	Chr	Start	End	Gene[1]	Known[2] (Yes/No)
All-cause dementia	1	2	127597930	128144615	*BIN1*	Yes
	2	6	40654030	41379252	*UNC5CL/TREML2/ TREM2*	Yes
	3	7	99527422	100234089	*SPDYE3/ZCWPW1/ NYAP1*	Yes
	4	8	27206253	27718503	*PTK2B/CLU*	Yes
	5	11	59690599	60273087	*MS4A4A/MS4A*	Yes
	6	19	44893942	45961598	*APOE*	Yes
Alzheimer's disease	1	2	127641427	128141427	*BIN1*	Yes
	2	6	40654030	41379252	*UNC5CL/TREML2/ TREM2*	Yes
	3	7	99527422	100234089	*SPDYE3/ZCWPW1/ NYAP1*	Yes
	4	15	63319902	63821820	*APH1B*	Yes
	5	19	44882679	45925180	*APOE*	Yes
Vascular dementia	1	1	10672576	11174273	*CASZ1*	No
	2	19	45074138	45677125	*APOE*	Yes

[1]Gene symbols from the meta-analysis of GWAS on Alzheimer's disease and related dementias by Bellenguez, C. *et al.* were used if the locus overlapped with their reports [1]. The gene symbol for the unreported locus was identified as the nearest gene to the top SNPs within the locus by gene annotation from the Ensembl database.
[2]Known loci were identified as overlapped with the loci reported by Bellenguez, C. *et al.*
Abbreviation: Chr, chromosome.

Table S5. Associations between FOS and the onset of all-cause dementia in genotype subgroups of SNPs in 43 loci and *APOE* ε4.

CHR	No. Loci	Start	End	SNP	POS	REF	ALT	HR of fish oil supplementation in participants carrying REF allele[1]						P-interaction
								0		1		2		
								HR (95%CI)	*p*-value	HR (95%CI)	*p*-value	HR (95%CI)	*p*-value	
19	-	-	-	*APOE* ε4	-	-	-	0.88 (0.81,0.94)	<0.001	0.93 (0.87,1.01)	0.082	0.97 (0.84,1.13)	0.701	0.029
1	1	8027198	8527198	rs116501531	8277198	A	G	0.91 (0.86,0.95)	<0.001	1.36 (1.03,1.81)	0.031	-	-	0.007
1	2	14816263	15316263	rs75837905	15066263	A	G	0.89 (0.85,0.94)	<0.001	1.44 (1.16,1.80)	0.001	-	-	<0.001
1	3	29232654	29732654	rs116264291	29482654	A	G	0.90 (0.86,0.95)	<0.001	1.42 (1.11,1.83)	0.006	-	-	<0.001
1	4	101852308	102352308	rs61804494	102102308	G	A	0.97 (0.91,1.03)	0.304	0.82 (0.74,0.90)	<0.001	0.62 (0.45,0.86)	0.004	<0.001
1	5	106483537	106983795	rs12043527	106733537	G	A	0.87 (0.83,0.92)	<0.001	1.10 (0.98,1.23)	0.108	1.20 (0.79,1.83)	0.395	<0.001
1	5	106483537	106983795	rs11184799	106733795	G	A	0.88 (0.83,0.93)	<0.001	1.05 (0.95,1.17)	0.316	1.12 (0.80,1.57)	0.526	<0.001
1	6	110597781	111098352	rs12024138	110847781	C	T	0.88 (0.84,0.93)	<0.001	1.29 (1.10,1.52)	0.002	1.43 (0.52,3.92)	0.485	<0.001
1	6	110597781	111098352	rs12024264	110848352	C	A	0.88 (0.84,0.93)	<0.001	1.29 (1.10,1.52)	0.002	1.43 (0.52,3.92)	0.485	<0.001
1	7	222375645	222876659	rs17163136	222625645	T	C	0.99 (0.92,1.07)	0.829	0.90 (0.84,0.97)	0.006	0.77 (0.68,0.88)	<0.001	0.002
1	7	222375645	222876659	rs17163137	222626659	T	C	0.99 (0.92,1.07)	0.831	0.90 (0.84,0.97)	0.005	0.78 (0.69,0.89)	<0.001	0.003
2	8	100571438	101071438	rs113777826	100821438	C	T	0.91 (0.86,0.95)	<0.001	1.28 (0.97,1.70)	0.08	-	-	0.017
2	9	170757664	171257664	rs4435418	171007664	C	T	0.81 (0.73,0.91)	<0.001	0.89 (0.83,0.95)	<0.001	1.05 (0.96,1.15)	0.284	<0.001
4	10	16312945	16812945	rs148811174	16562945	C	T	0.98 (0.92,1.04)	0.421	0.79 (0.73,0.87)	<0.001	0.94 (0.73,1.21)	0.651	0.003
4	11	68145282	68645282	rs149325653	68395282	A	G	0.93 (0.88,0.97)	0.003	0.66 (0.48,0.91)	0.011	-	-	0.037
4	12	114132769	114635835	rs13123494	114382769	C	A	0.85 (0.79,0.92)	<0.001	0.93 (0.86,0.99)	0.036	1.03 (0.91,1.17)	0.623	0.009
4	12	114132769	114635835	rs1525000	114384869	C	T	0.85 (0.78,0.92)	<0.001	0.93 (0.87,1.00)	0.048	1.02 (0.90,1.15)	0.794	0.011

4	12	114132769	114635835	rs62314976	114385676	T	C	0.85 (0.78,0.92)	<0.001	0.93 (0.87,1.00)	0.054	1.02 (0.90,1.16)	0.72	0.008
4	12	114132769	114635835	rs13106836	114385835	G	A	0.85 (0.78,0.92)	<0.001	0.93 (0.87,1.00)	0.054	1.02 (0.90,1.15)	0.734	0.008
5	13	3009680	3517512	rs7722735	3259680	G	T	0.96 (0.90,1.03)	0.268	0.91 (0.85,0.98)	0.016	0.73 (0.62,0.86)	<0.001	0.006
5	13	3009680	3517512	rs59781823	3262724	G	A	0.96 (0.89,1.03)	0.265	0.91 (0.85,0.98)	0.018	0.72 (0.61,0.85)	<0.001	0.004
5	13	3009680	3517512	rs60808439	3267512	C	T	0.95 (0.89,1.01)	0.088	0.89 (0.82,0.97)	0.008	0.73 (0.57,0.93)	0.012	0.052
5	14	100147117	100647117	rs2089903	100397117	C	A	-	-	1.39 (1.10,1.74)	0.005	0.90 (0.85,0.94)	<0.001	<0.001
6	15	767009	1267009	rs845890	1017009	C	A	0.89 (0.84,0.95)	<0.001	0.95 (0.87,1.03)	0.21	0.98 (0.77,1.24)	0.844	0.238
6	16	115069829	115569829	rs9505681	115319829	C	T	0.90 (0.85,0.94)	<0.001	1.61 (1.21,2.15)	0.001	-	-	<0.001
6	17	166801161	167302048	rs6941412	167051161	C	T	1.01 (0.94,1.07)	0.881	0.85 (0.78,0.92)	<0.001	0.70 (0.57,0.84)	<0.001	<0.001
6	17	166801161	167302048	rs12174679	167051368	G	A	1.00 (0.94,1.07)	0.885	0.84 (0.78,0.91)	<0.001	0.71 (0.58,0.87)	<0.001	<0.001
6	17	166801161	167302048	rs963302	167052048	C	G	1.01 (0.94,1.08)	0.859	0.86 (0.79,0.93)	<0.001	0.68 (0.57,0.82)	<0.001	<0.001
7	18	20096169	20596169	rs3114430	20346169	C	A	1.13 (0.98,1.30)	0.088	0.95 (0.88,1.03)	0.192	0.81 (0.75,0.88)	<0.001	<0.001
7	19	143574310	144074310	rs12670543	143824310	C	A	0.86 (0.82,0.91)	<0.001	1.17 (1.04,1.31)	0.007	1.23 (0.74,2.04)	0.417	<0.001
8	20	3675912	4175912	rs2042528	3925912	A	T	0.99 (0.90,1.09)	0.833	0.93 (0.87,1.00)	0.058	0.80 (0.72,0.89)	<0.001	0.005
8	21	82617116	83117116	rs117216345	82867116	T	A	0.91 (0.86,0.96)	<0.001	0.99 (0.87,1.13)	0.865	1.00 (0.55,1.85)	0.989	0.242
8	22	111221071	111721071	rs116871946	111471071	T	C	0.93 (0.89,0.98)	0.009	0.76 (0.64,0.90)	0.001	0.58 (0.25,1.36)	0.211	0.01
10	23	4149849	4649849	rs116876958	4399849	C	T	0.91 (0.86,0.96)	<0.001	1.03 (0.84,1.26)	0.785	-	-	0.165
10	24	129615006	130115006	rs4750683	129865006	G	C	1.05 (0.87,1.26)	0.61	1.02 (0.94,1.11)	0.593	0.84 (0.79,0.90)	<0.001	<0.001
11	25	85731587	86233703	rs75559794	85981587	G	A	0.89 (0.85,0.94)	<0.001	1.06 (0.93,1.21)	0.363	1.26 (0.67,2.37)	0.471	0.01
11	25	85731587	86233703	rs78851816	85983703	G	A	0.89 (0.85,0.94)	<0.001	1.06 (0.93,1.21)	0.394	1.28 (0.68,2.40)	0.45	0.01
11	26	96280700	96825966	rs754413	96530700	A	G	1.54 (1.03,2.29)	0.035	1.04 (0.93,1.15)	0.512	0.88 (0.83,0.93)	<0.001	<0.001
11	26	96280700	96825966	rs3018644	96575966	G	A	1.45 (0.96,2.18)	0.077	1.06 (0.95,1.17)	0.287	0.87 (0.82,0.92)	<0.001	<0.001

12	27	47212480	47712480	rs74523587	47462480	A	C	0.90 (0.85,0.94)	<0.001	1.40 (1.12,1.76)	0.003	-	-	<0.001
12	28	55968568	56468568	rs73119275	56218568	C	T	0.90 (0.85,0.94)	<0.001	1.56 (1.22,2.00)	<0.001	-	-	<0.001
12	29	69525118	70042102	rs2870901	69775118	C	T	0.80 (0.68,0.94)	0.006	0.90 (0.83,0.97)	0.004	0.96 (0.89,1.03)	0.21	0.024
12	29	69525118	70042102	rs1585705	69792102	A	C	0.77 (0.65,0.90)	<0.001	0.91 (0.84,0.98)	0.011	0.96 (0.89,1.03)	0.265	0.012
12	30	104063861	104572214	rs11111842	104313861	A	G	0.91 (0.86,0.96)	<0.001	0.96 (0.83,1.10)	0.546	1.13 (0.44,2.88)	0.803	0.494
12	30	104063861	104572214	rs10861145	104314531	G	C	0.91 (0.86,0.96)	<0.001	0.96 (0.83,1.10)	0.545	1.13 (0.44,2.88)	0.803	0.495
12	30	104063861	104572214	rs11833702	104314560	A	G	0.91 (0.86,0.96)	<0.001	0.96 (0.84,1.11)	0.58	1.13 (0.44,2.88)	0.805	0.463
12	30	104063861	104572214	rs11111843	104314956	T	C	0.91 (0.86,0.96)	<0.001	0.96 (0.83,1.10)	0.545	1.13 (0.44,2.88)	0.803	0.498
12	30	104063861	104572214	rs11111844	104316005	T	C	0.91 (0.86,0.96)	<0.001	0.96 (0.84,1.10)	0.566	1.13 (0.44,2.88)	0.804	0.477
12	30	104063861	104572214	rs76025677	104317050	A	G	0.91 (0.86,0.96)	<0.001	0.96 (0.84,1.11)	0.6	1.13 (0.44,2.88)	0.804	0.45
12	30	104063861	104572214	rs11111846	104318346	T	C	0.91 (0.86,0.96)	<0.001	0.96 (0.84,1.10)	0.566	1.13 (0.44,2.88)	0.804	0.47
12	30	104063861	104572214	rs11111849	104319873	A	G	0.91 (0.86,0.96)	<0.001	0.96 (0.84,1.10)	0.566	1.13 (0.44,2.88)	0.804	0.475
12	30	104063861	104572214	rs11111850	104321073	A	T	0.91 (0.86,0.96)	<0.001	0.96 (0.84,1.10)	0.566	1.13 (0.44,2.88)	0.804	0.474
12	30	104063861	104572214	rs3794246	104321902	T	C	0.91 (0.86,0.96)	<0.001	0.96 (0.83,1.10)	0.541	1.13 (0.44,2.89)	0.8	0.495
12	30	104063861	104572214	rs17034916	104322214	C	T	0.91 (0.86,0.96)	<0.001	0.96 (0.84,1.10)	0.566	1.13 (0.44,2.88)	0.804	0.474
13	31	26140990	26728255	rs117674980	26390990	A	G	0.92 (0.88,0.97)	0.001	0.83 (0.68,1.01)	0.056	0.31 (0.06,1.62)	0.165	0.186
13	31	26140990	26728255	rs77999054	26394850	G	A	0.92 (0.88,0.97)	0.001	0.84 (0.69,1.02)	0.072	0.31 (0.06,1.62)	0.165	0.222
13	31	26140990	26728255	rs3783124	26458231	C	T	0.92 (0.88,0.97)	0.001	0.85 (0.70,1.04)	0.108	0.06 (0.00,2.91)	0.153	0.27
13	31	26140990	26728255	rs74335017	26478255	C	T	0.92 (0.88,0.97)	0.001	0.87 (0.72,1.06)	0.172	0.03 (0.00,1.41)	0.075	0.359
13	32	67331127	67831127	rs9571707	67581127	A	G	0.85 (0.79,0.91)	<0.001	0.96 (0.89,1.04)	0.313	1.11 (0.93,1.31)	0.247	<0.001
13	33	93315090	93875433	rs1932193	93565090	A	C	0.85 (0.78,0.93)	<0.001	0.95 (0.89,1.02)	0.179	0.95 (0.85,1.06)	0.364	0.081
13	33	93315090	93875433	rs306675	93606083	A	C	0.83 (0.74,0.93)	0.002	0.94 (0.88,1.01)	0.092	0.93 (0.86,1.02)	0.125	0.161

13	33	93315090	93875433	rs306677	93613362	C	G	0.83 (0.74,0.94)	0.003	0.94 (0.88,1.01)	0.087	0.94 (0.86,1.02)	0.134	0.169
13	33	93315090	93875433	rs306679	93625433	G	T	0.84 (0.74,0.95)	0.006	0.95 (0.88,1.02)	0.157	0.93 (0.85,1.01)	0.08	0.302
13	34	100252104	100752104	rs72653992	100502104	A	G	0.90 (0.86,0.95)	<0.001	1.10 (0.92,1.32)	0.298	0.73 (0.22,2.44)	0.61	0.039
14	35	57055847	57555847	rs12896185	57305847	A	G	0.85 (0.80,0.91)	<0.001	0.99 (0.91,1.08)	0.829	1.23 (1.00,1.51)	0.052	<0.001
14	36	80321393	80823172	rs1181351	80571393	G	T	0.86 (0.71,1.03)	0.094	0.95 (0.88,1.02)	0.169	0.89 (0.83,0.96)	0.001	0.657
14	36	80321393	80823172	rs28444185	80573172	A	T	0.89 (0.83,0.95)	<0.001	0.95 (0.88,1.03)	0.204	0.86 (0.72,1.03)	0.103	0.575
15	37	90077226	90587639	rs28698386	90327226	T	C	1.01 (0.91,1.11)	0.884	0.91 (0.85,0.97)	0.007	0.86 (0.78,0.95)	0.002	0.02
15	37	90077226	90587639	rs6496605	90337639	C	T	1.00 (0.91,1.11)	0.939	0.90 (0.84,0.97)	0.004	0.85 (0.77,0.94)	0.001	0.016
16	38	13669429	14169429	rs75422462	13919429	G	A	0.91 (0.86,0.95)	<0.001	1.17 (0.91,1.51)	0.226	-	-	0.029
18	39	52608233	53108233	rs34352315	52858233	G	A	0.93 (0.89,0.98)	0.004	0.53 (0.38,0.73)	<0.001	-	-	<0.001
21	40	17158456	17658456	rs2823575	17408456	G	A	0.89 (0.84,0.94)	<0.001	1.05 (0.94,1.17)	0.408	0.63 (0.39,1.03)	0.064	0.099
21	41	40203038	40703038	rs74482819	40453038	C	T	0.90 (0.86,0.95)	<0.001	1.33 (1.04,1.69)	0.021	-	-	0.004
22	42	18315772	18842083	rs71328255	18565772	A	G	0.90 (0.85,0.94)	<0.001	1.57 (1.21,2.05)	<0.001	-	-	<0.001
22	42	18315772	18842083	rs71328256	18569926	T	C	0.90 (0.85,0.94)	<0.001	1.57 (1.21,2.05)	<0.001	-	-	<0.001
22	42	18315772	18842083	rs34680388	18576176	T	A	0.90 (0.85,0.94)	<0.001	1.57 (1.21,2.04)	<0.001	-	-	<0.001
22	42	18315772	18842083	rs13058445	18580087	A	G	0.90 (0.85,0.94)	<0.001	1.57 (1.20,2.04)	<0.001	-	-	<0.001
22	42	18315772	18842083	rs34658760	18584324	A	C	0.90 (0.85,0.94)	<0.001	1.58 (1.21,2.06)	<0.001	-	-	<0.001
22	42	18315772	18842083	rs34667409	18584379	A	G	0.90 (0.85,0.94)	<0.001	1.57 (1.21,2.04)	<0.001	-	-	<0.001
22	42	18315772	18842083	rs35137695	18587898	C	T	0.90 (0.85,0.94)	<0.001	1.59 (1.22,2.07)	<0.001	-	-	<0.001
22	42	18315772	18842083	rs13058179	18592083	G	A	0.90 (0.85,0.94)	<0.001	1.54 (1.18,2.00)	0.001	-	-	<0.001
22	43	29569106	30069106	rs144548824	29819106	G	A	0.94 (0.89,0.99)	0.011	0.59 (0.47,0.75)	<0.001	-	-	<0.001

[1]The risk of fish oil supplementation for the development of all-cause dementia was evaluated using Cox regression models adjusted by age, sex, and top 10 genetic principal components within each SNP genotype. Genotype groups with five or fewer incident cases were not considered in the subgroup analysis.

Abbreviations: ALT, alternative allele; CHR, chromosome; CI, confidence interval; HR, hazard ratio; POS, SNP position with GRCh37 assembly; P-interaction, *p*-value of the interaction terms; REF, reference allele; SNP, single nucleotide polymorphism.

Table S6. Associations between FOS and the onset of Alzheimer's disease in genotype subgroups of SNPs in 43 loci and *APOE* ε4.

CHR	No. Loci	Start	End	SNP	POS	REF	ALT	HR of fish oil supplementation in participants carrying REF allele[1]						P-interaction
								0		1		2		
								HR (95%CI)	*p*-value	HR (95%CI)	*p*-value	HR (95%CI)	*p*-value	
19	-	-	-	*APOE* ε4	-	-	-	1.01 (0.90,1.14)	0.821	0.99 (0.89,1.10)	0.911	1.03 (0.85,1.25)	0.752	0.912
1	1	8027198	8527198	rs116501531	8277198	A	G	0.98 (0.91,1.05)	0.57	2.18 (1.48,3.20)	<0.001	-	-	<0.001
1	2	14816263	15316263	rs75837905	15066263	A	G	0.97 (0.90,1.04)	0.42	1.95 (1.42,2.66)	<0.001	-	-	<0.001
1	3	29232654	29732654	rs116264291	29482654	A	G	0.99 (0.92,1.07)	0.815	1.58 (1.06,2.35)	0.026	-	-	0.012
1	4	101852308	102352308	rs61804494	102102308	G	A	1.08 (0.99,1.18)	0.069	0.85 (0.74,0.98)	0.029	0.50 (0.28,0.89)	0.018	<0.001
1	5	106483537	106983795	rs12043527	106733537	G	A	0.97 (0.90,1.05)	0.479	1.17 (0.99,1.40)	0.073	1.51 (0.82,2.78)	0.182	0.012
1	5	106483537	106983795	rs11184799	106733795	G	A	0.99 (0.91,1.08)	0.811	1.10 (0.94,1.29)	0.214	1.36 (0.85,2.17)	0.206	0.076
1	6	110597781	111098352	rs12024138	110847781	C	T	0.96 (0.89,1.03)	0.271	1.63 (1.28,2.06)	<0.001	1.44 (0.31,6.57)	0.639	<0.001
1	6	110597781	111098352	rs12024264	110848352	C	A	0.96 (0.89,1.03)	0.271	1.63 (1.28,2.06)	<0.001	1.44 (0.31,6.57)	0.639	<0.001
1	7	222375645	222876659	rs17163136	222625645	T	C	1.08 (0.96,1.21)	0.212	1.01 (0.91,1.12)	0.873	0.83 (0.69,1.01)	0.068	0.056
1	7	222375645	222876659	rs17163137	222626659	T	C	1.08 (0.96,1.21)	0.219	1.01 (0.91,1.12)	0.883	0.84 (0.69,1.02)	0.079	0.065
2	8	100571438	101071438	rs113777826	100821438	C	T	1.01 (0.94,1.09)	0.77	1.04 (0.67,1.63)	0.86	-	-	0.851
2	9	170757664	171257664	rs4435418	171007664	C	T	0.93 (0.79,1.10)	0.392	0.96 (0.87,1.07)	0.45	1.14 (1.00,1.30)	0.057	0.038
4	10	16312945	16812945	rs148811174	16562945	C	T	1.03 (0.94,1.13)	0.485	0.93 (0.82,1.06)	0.303	1.19 (0.82,1.72)	0.364	0.675
4	11	68145282	68645282	rs149325653	68395282	A	G	1.04 (0.97,1.12)	0.25	0.40 (0.24,0.65)	<0.001	-	-	<0.001
4	12	114132769	114635835	rs13123494	114382769	C	A	1.01 (0.89,1.13)	0.921	1.01 (0.91,1.12)	0.862	1.02 (0.85,1.23)	0.801	0.874
4	12	114132769	114635835	rs1525000	114384869	C	T	1.00 (0.89,1.13)	0.988	1.01 (0.91,1.13)	0.806	1.02 (0.85,1.22)	0.871	0.881
4	12	114132769	114635835	rs62314976	114385676	T	C	0.99 (0.88,1.12)	0.916	1.02 (0.92,1.14)	0.691	1.01 (0.84,1.21)	0.916	0.811

4	12	114132769	114635835	rs13106836	114385835	G	A	0.99 (0.88,1.12)	0.924	1.02 (0.92,1.14)	0.694	1.01 (0.84,1.21)	0.915	0.813
5	13	3009680	3517512	rs7722735	3259680	G	T	1.14 (1.03,1.27)	0.013	0.96 (0.86,1.07)	0.495	0.69 (0.55,0.88)	0.002	<0.001
5	13	3009680	3517512	rs59781823	3262724	G	A	1.14 (1.03,1.27)	0.014	0.96 (0.86,1.08)	0.511	0.68 (0.54,0.87)	0.002	<0.001
5	13	3009680	3517512	rs60808439	3267512	C	T	1.12 (1.02,1.23)	0.023	0.91 (0.80,1.03)	0.119	0.62 (0.43,0.89)	0.01	<0.001
5	14	100147117	100647117	rs2089903	100397117	C	A	-	-	1.56 (1.11,2.19)	0.011	0.99 (0.92,1.06)	0.763	0.029
6	15	767009	1267009	rs845890	1017009	C	A	0.99 (0.90,1.08)	0.814	1.03 (0.90,1.17)	0.67	1.10 (0.75,1.60)	0.627	0.389
6	16	115069829	115569829	rs9505681	115319829	C	T	0.98 (0.91,1.06)	0.667	2.08 (1.31,3.29)	0.002	-	-	0.001
6	17	166801161	167302048	rs6941412	167051161	C	T	1.08 (0.98,1.19)	0.118	0.96 (0.86,1.09)	0.541	0.75 (0.56,0.99)	0.043	0.006
6	17	166801161	167302048	rs12174679	167051368	G	A	1.09 (0.99,1.20)	0.087	0.94 (0.84,1.06)	0.344	0.79 (0.59,1.06)	0.122	0.007
6	17	166801161	167302048	rs963302	167052048	C	G	1.08 (0.98,1.19)	0.107	0.97 (0.86,1.09)	0.575	0.73 (0.55,0.97)	0.027	0.004
7	18	20096169	20596169	rs3114430	20346169	C	A	1.20 (0.97,1.48)	0.092	1.00 (0.90,1.12)	0.983	0.95 (0.85,1.06)	0.339	0.078
7	19	143574310	144074310	rs12670543	143824310	C	A	0.96 (0.88,1.04)	0.269	1.24 (1.05,1.48)	0.013	1.29 (0.58,2.89)	0.53	0.008
8	20	3675912	4175912	rs2042528	3925912	A	T	1.23 (1.07,1.41)	0.004	1.01 (0.91,1.12)	0.866	0.80 (0.68,0.94)	0.007	<0.001
8	21	82617116	83117116	rs117216345	82867116	T	A	1.03 (0.95,1.12)	0.431	0.97 (0.80,1.18)	0.753	0.64 (0.20,2.00)	0.439	0.334
8	22	111221071	111721071	rs116871946	111471071	T	C	1.05 (0.97,1.13)	0.198	0.68 (0.53,0.87)	0.002	0.10 (0.01,0.85)	0.035	<0.001
10	23	4149849	4649849	rs116876958	4399849	C	T	1.01 (0.94,1.09)	0.806	0.96 (0.70,1.33)	0.819	-	-	0.991
10	24	129615006	130115006	rs4750683	129865006	G	C	1.14 (0.87,1.49)	0.334	1.16 (1.03,1.31)	0.015	0.91 (0.82,1.00)	0.056	0.005
11	25	85731587	86233703	rs75559794	85981587	G	A	0.94 (0.87,1.02)	0.118	1.48 (1.23,1.79)	<0.001	1.48 (0.66,3.35)	0.342	<0.001
11	25	85731587	86233703	rs78851816	85983703	G	A	0.94 (0.87,1.01)	0.11	1.49 (1.24,1.79)	<0.001	1.50 (0.67,3.39)	0.328	<0.001
11	26	96280700	96825966	rs754413	96530700	A	G	1.46 (0.83,2.57)	0.19	1.20 (1.03,1.41)	0.022	0.95 (0.88,1.03)	0.254	0.003
11	26	96280700	96825966	rs3018644	96575966	G	A	1.17 (0.64,2.15)	0.602	1.22 (1.04,1.42)	0.013	0.95 (0.88,1.04)	0.259	0.006
12	27	47212480	47712480	rs74523587	47462480	A	C	0.99 (0.92,1.07)	0.882	1.42 (1.02,1.97)	0.037	-	-	0.051

12	28	55968568	56468568	rs73119275	56218568	C	T	0.98 (0.91,1.06)	0.671	1.86 (1.28,2.70)	0.001	-	-	<0.001
12	29	69525118	70042102	rs2870901	69775118	C	T	0.90 (0.71,1.13)	0.364	1.04 (0.93,1.17)	0.467	1.00 (0.90,1.11)	0.998	0.634
12	29	69525118	70042102	rs1585705	69792102	A	C	0.78 (0.62,0.99)	0.038	1.04 (0.93,1.16)	0.504	1.03 (0.93,1.14)	0.582	0.116
12	30	104063861	104572214	rs11111842	104313861	A	G	1.01 (0.93,1.09)	0.837	1.02 (0.83,1.25)	0.854	0.40 (0.04,3.56)	0.409	0.887
12	30	104063861	104572214	rs10861145	104314531	G	C	1.01 (0.93,1.09)	0.836	1.02 (0.83,1.25)	0.855	0.40 (0.04,3.56)	0.409	0.886
12	30	104063861	104572214	rs11833702	104314560	A	G	1.01 (0.93,1.09)	0.826	1.02 (0.83,1.25)	0.857	0.40 (0.04,3.56)	0.409	0.882
12	30	104063861	104572214	rs11111843	104314956	T	C	1.01 (0.93,1.09)	0.825	1.02 (0.83,1.25)	0.855	0.40 (0.04,3.56)	0.409	0.881
12	30	104063861	104572214	rs11111844	104316005	T	C	1.01 (0.93,1.09)	0.825	1.02 (0.83,1.25)	0.852	0.40 (0.04,3.56)	0.409	0.884
12	30	104063861	104572214	rs76025677	104317050	A	G	1.01 (0.93,1.09)	0.835	1.02 (0.83,1.26)	0.824	0.40 (0.04,3.56)	0.409	0.915
12	30	104063861	104572214	rs11111846	104318346	T	C	1.01 (0.93,1.09)	0.855	1.02 (0.83,1.25)	0.851	0.40 (0.04,3.56)	0.409	0.896
12	30	104063861	104572214	rs11111849	104319873	A	G	1.01 (0.93,1.09)	0.836	1.02 (0.83,1.25)	0.852	0.40 (0.04,3.56)	0.409	0.889
12	30	104063861	104572214	rs11111850	104321073	A	T	1.01 (0.93,1.09)	0.836	1.02 (0.83,1.25)	0.852	0.40 (0.04,3.56)	0.409	0.889
12	30	104063861	104572214	rs3794246	104321902	T	C	1.01 (0.93,1.09)	0.874	1.02 (0.83,1.25)	0.857	0.40 (0.04,3.56)	0.409	0.898
12	30	104063861	104572214	rs17034916	104322214	C	T	1.01 (0.93,1.09)	0.836	1.02 (0.83,1.25)	0.852	0.40 (0.04,3.56)	0.409	0.889
13	31	26140990	26728255	rs117674980	26390990	A	G	1.00 (0.93,1.08)	0.906	1.03 (0.78,1.36)	0.835	-	-	0.993
13	31	26140990	26728255	rs77999054	26394850	G	A	1.01 (0.93,1.08)	0.87	1.03 (0.78,1.36)	0.848	-	-	0.988
13	31	26140990	26728255	rs3783124	26458231	C	T	1.00 (0.93,1.08)	0.9	1.06 (0.80,1.41)	0.681	-	-	0.831
13	31	26140990	26728255	rs74335017	26478255	C	T	1.01 (0.93,1.08)	0.874	1.09 (0.82,1.46)	0.546	-	-	0.723
13	32	67331127	67831127	rs9571707	67581127	A	G	0.85 (0.77,0.94)	0.002	1.14 (1.02,1.27)	0.022	1.42 (1.11,1.81)	0.006	<0.001
13	33	93315090	93875433	rs1932193	93565090	A	C	0.82 (0.72,0.93)	0.003	1.05 (0.95,1.17)	0.349	1.18 (1.01,1.38)	0.042	<0.001
13	33	93315090	93875433	rs306675	93606083	A	C	0.82 (0.69,0.98)	0.026	0.94 (0.84,1.04)	0.238	1.25 (1.10,1.41)	<0.001	<0.001
13	33	93315090	93875433	rs306677	93613362	C	G	0.83 (0.69,0.98)	0.033	0.94 (0.84,1.04)	0.243	1.25 (1.10,1.41)	<0.001	<0.001

13	33	93315090	93875433	rs306679	93625433	G	T	0.80 (0.66,0.96)	0.016	0.96 (0.86,1.07)	0.451	1.23 (1.09,1.39)	<0.001	<0.001
13	34	100252104	100752104	rs72653992	100502104	A	G	0.99 (0.92,1.07)	0.884	1.18 (0.90,1.55)	0.226	-	-	0.15
14	35	57055847	57555847	rs12896185	57305847	A	G	0.95 (0.87,1.04)	0.283	1.09 (0.96,1.23)	0.189	1.18 (0.87,1.61)	0.291	0.047
14	36	80321393	80823172	rs1181351	80571393	G	T	0.90 (0.68,1.18)	0.437	1.03 (0.92,1.16)	0.569	1.00 (0.90,1.10)	0.963	0.919
14	36	80321393	80823172	rs28444185	80573172	A	T	0.99 (0.90,1.10)	0.887	1.03 (0.92,1.16)	0.567	0.89 (0.68,1.18)	0.414	0.955
15	37	90077226	90587639	rs28698386	90327226	T	C	1.30 (1.13,1.50)	<0.001	0.95 (0.86,1.05)	0.323	0.88 (0.75,1.01)	0.077	<0.001
15	37	90077226	90587639	rs6496605	90337639	C	T	1.29 (1.12,1.48)	<0.001	0.95 (0.86,1.05)	0.328	0.87 (0.75,1.01)	0.064	<0.001
16	38	13669429	14169429	rs75422462	13919429	G	A	0.98 (0.91,1.06)	0.61	1.86 (1.30,2.67)	<0.001	-	-	<0.001
18	39	52608233	53108233	rs34352315	52858233	G	A	1.04 (0.97,1.12)	0.273	0.36 (0.22,0.60)	<0.001	-	-	<0.001
21	40	17158456	17658456	rs2823575	17408456	G	A	1.00 (0.92,1.08)	0.948	1.08 (0.92,1.28)	0.328	0.61 (0.31,1.21)	0.156	0.812
21	41	40203038	40703038	rs74482819	40453038	C	T	1.00 (0.93,1.07)	0.91	1.42 (0.97,2.08)	0.073	-	-	0.121
22	42	18315772	18842083	rs71328255	18565772	A	G	0.99 (0.92,1.06)	0.731	1.67 (1.13,2.45)	0.01	-	-	<0.001
22	42	18315772	18842083	rs71328256	18569926	T	C	0.99 (0.92,1.06)	0.747	1.66 (1.13,2.45)	0.01	-	-	<0.001
22	42	18315772	18842083	rs34680388	18576176	T	A	0.99 (0.92,1.06)	0.743	1.65 (1.12,2.43)	0.011	-	-	0.002
22	42	18315772	18842083	rs13058445	18580087	A	G	0.99 (0.92,1.06)	0.742	1.66 (1.12,2.44)	0.011	-	-	<0.001
22	42	18315772	18842083	rs34658760	18584324	A	C	0.99 (0.92,1.06)	0.742	1.66 (1.13,2.44)	0.011	-	-	<0.001
22	42	18315772	18842083	rs34667409	18584379	A	G	0.99 (0.92,1.06)	0.743	1.66 (1.13,2.44)	0.011	-	-	<0.001
22	42	18315772	18842083	rs35137695	18587898	C	T	0.99 (0.92,1.06)	0.744	1.65 (1.12,2.44)	0.011	-	-	0.001
22	42	18315772	18842083	rs13058179	18592083	G	A	0.99 (0.92,1.06)	0.747	1.58 (1.07,2.33)	0.021	-	-	0.002
22	43	29569106	30069106	rs144548824	29819106	G	A	1.04 (0.97,1.12)	0.265	0.52 (0.36,0.74)	<0.001	-	-	<0.001

[1]The risk of fish oil supplementation for the development of Alzheimer's disease was evaluated using Cox regression models adjusted by age, sex, and top 10 genetic principal components within each SNP genotype. Genotype groups with five or fewer incident cases were not considered in the subgroup analysis.

Abbreviations: ALT, alternative allele; CHR, chromosome; CI, confidence interval; HR, hazard ratio; POS, SNP position with GRCh37 assembly; P-interaction, *p*-value of the interaction terms; REF, reference allele; SNP, single nucleotide polymorphism.

Table S7. Associations between FOS and the onset of vascular dementia in genotype subgroups of SNPs in 43 loci and *APOE* ε4.

CHR	No. Loci	Start	End	SNP	POS	REF	ALT	HR of fish oil supplementation in participants carrying REF allele[1]						P-interaction
								0		1		2		
								HR (95%CI)	*p*-value	HR (95%CI)	*p*-value	HR (95%CI)	*p*-value	
19	-	-	-	*APOE* ε4	-	-	-	0.74 (0.64,0.86)	<0.001	0.80 (0.68,0.95)	0.011	1.21 (0.87,1.69)	0.264	0.009
1	1	8027198	8527198	rs116501531	8277198	A	G	0.80 (0.71,0.89)	<0.001	1.10 (0.59,2.05)	0.767	-	-	0.413
1	2	14816263	15316263	rs75837905	15066263	A	G	0.79 (0.71,0.88)	<0.001	1.18 (0.76,1.84)	0.463	-	-	0.145
1	3	29232654	29732654	rs116264291	29482654	A	G	0.78 (0.70,0.87)	<0.001	1.47 (0.88,2.46)	0.138	-	-	0.009
1	4	101852308	102352308	rs61804494	102102308	G	A	0.87 (0.77,0.98)	0.024	0.69 (0.55,0.85)	<0.001	0.41 (0.18,0.94)	0.035	0.009
1	5	106483537	106983795	rs12043527	106733537	G	A	0.73 (0.65,0.82)	<0.001	1.13 (0.90,1.42)	0.286	1.02 (0.44,2.37)	0.964	<0.001
1	5	106483537	106983795	rs11184799	106733795	G	A	0.70 (0.62,0.80)	<0.001	1.09 (0.89,1.34)	0.393	1.25 (0.63,2.46)	0.529	<0.001
1	6	110597781	111098352	rs12024138	110847781	C	T	0.77 (0.69,0.86)	<0.001	1.25 (0.87,1.78)	0.225	1.33 (0.25,7.04)	0.739	0.006
1	6	110597781	111098352	rs12024264	110848352	C	A	0.77 (0.69,0.86)	<0.001	1.25 (0.87,1.78)	0.224	1.33 (0.25,7.04)	0.739	0.005
1	7	222375645	222876659	rs17163136	222625645	T	C	1.04 (0.88,1.22)	0.68	0.72 (0.62,0.84)	<0.001	0.57 (0.43,0.76)	<0.001	<0.001
1	7	222375645	222876659	rs17163137	222626659	T	C	1.04 (0.88,1.23)	0.653	0.72 (0.61,0.84)	<0.001	0.57 (0.43,0.76)	<0.001	<0.001
2	8	100571438	101071438	rs113777826	100821438	C	T	0.77 (0.69,0.86)	<0.001	2.16 (1.27,3.68)	0.004	-	-	<0.001
2	9	170757664	171257664	rs4435418	171007664	C	T	0.75 (0.60,0.94)	0.012	0.85 (0.73,0.99)	0.035	0.83 (0.68,1.01)	0.058	0.58
4	10	16312945	16812945	rs148811174	16562945	C	T	0.99 (0.87,1.12)	0.846	0.53 (0.44,0.65)	<0.001	0.48 (0.26,0.87)	0.016	<0.001
4	11	68145282	68645282	rs149325653	68395282	A	G	0.80 (0.72,0.89)	<0.001	0.82 (0.39,1.71)	0.602	-	-	0.84
4	12	114132769	114635835	rs13123494	114382769	C	A	0.63 (0.52,0.76)	<0.001	0.79 (0.68,0.93)	0.003	1.30 (1.02,1.66)	0.034	<0.001
4	12	114132769	114635835	rs1525000	114384869	C	T	0.63 (0.52,0.76)	<0.001	0.80 (0.69,0.93)	0.004	1.26 (0.99,1.61)	0.058	<0.001
4	12	114132769	114635835	rs62314976	114385676	T	C	0.63 (0.52,0.76)	<0.001	0.80 (0.69,0.93)	0.005	1.29 (1.01,1.65)	0.04	<0.001

4	12	114132769	114635835	rs13106836	114385835	G	A	0.63 (0.52,0.75)	<0.001	0.80 (0.69,0.93)	0.005	1.29 (1.01,1.65)	0.04	<0.001
5	13	3009680	3517512	rs7722735	3259680	G	T	0.80 (0.69,0.93)	0.003	0.81 (0.69,0.96)	0.012	0.78 (0.55,1.11)	0.169	0.953
5	13	3009680	3517512	rs59781823	3262724	G	A	0.79 (0.68,0.92)	0.002	0.83 (0.70,0.97)	0.02	0.74 (0.52,1.05)	0.094	0.905
5	13	3009680	3517512	rs60808439	3267512	C	T	0.80 (0.70,0.92)	0.001	0.79 (0.66,0.95)	0.013	0.90 (0.53,1.53)	0.701	0.94
5	14	100147117	100647117	rs2089903	100397117	C	A	-	-	2.56 (1.59,4.11)	<0.001	0.75 (0.68,0.84)	<0.001	<0.001
6	15	767009	1267009	rs845890	1017009	C	A	0.68 (0.59,0.78)	<0.001	0.98 (0.82,1.17)	0.788	1.35 (0.85,2.13)	0.205	<0.001
6	16	115069829	115569829	rs9505681	115319829	C	T	0.79 (0.71,0.88)	<0.001	1.31 (0.64,2.70)	0.466	-	-	0.137
6	17	166801161	167302048	rs6941412	167051161	C	T	0.89 (0.77,1.02)	0.104	0.71 (0.60,0.85)	<0.001	0.64 (0.42,0.98)	0.04	0.02
6	17	166801161	167302048	rs12174679	167051368	G	A	0.89 (0.77,1.02)	0.089	0.72 (0.60,0.85)	<0.001	0.62 (0.40,0.96)	0.031	0.02
6	17	166801161	167302048	rs963302	167052048	C	G	0.88 (0.76,1.01)	0.079	0.73 (0.61,0.86)	<0.001	0.65 (0.43,0.97)	0.034	0.033
7	18	20096169	20596169	rs3114430	20346169	C	A	0.97 (0.71,1.31)	0.826	0.86 (0.73,1.01)	0.065	0.71 (0.61,0.84)	<0.001	0.069
7	19	143574310	144074310	rs12670543	143824310	C	A	0.75 (0.67,0.85)	<0.001	1.07 (0.83,1.38)	0.61	1.54 (0.48,4.97)	0.469	0.009
8	20	3675912	4175912	rs2042528	3925912	A	T	0.81 (0.67,0.99)	0.039	0.89 (0.77,1.04)	0.141	0.66 (0.53,0.84)	<0.001	0.341
8	21	82617116	83117116	rs117216345	82867116	T	A	0.73 (0.65,0.83)	<0.001	1.27 (0.97,1.66)	0.079	1.75 (0.70,4.39)	0.231	<0.001
8	22	111221071	111721071	rs116871946	111471071	T	C	0.82 (0.73,0.91)	<0.001	0.65 (0.46,0.93)	0.018	2.28 (0.43,12.17)	0.335	0.714
10	23	4149849	4649849	rs116876958	4399849	C	T	0.76 (0.68,0.85)	<0.001	1.63 (1.11,2.40)	0.012	-	-	<0.001
10	24	129615006	130115006	rs4750683	129865006	G	C	0.78 (0.52,1.17)	0.226	0.83 (0.70,0.99)	0.036	0.78 (0.68,0.90)	<0.001	0.793
11	25	85731587	86233703	rs75559794	85981587	G	A	0.79 (0.71,0.89)	<0.001	0.89 (0.66,1.19)	0.422	-	-	0.474
11	25	85731587	86233703	rs78851816	85983703	G	A	0.80 (0.71,0.89)	<0.001	0.85 (0.64,1.13)	0.266	-	-	0.636
11	26	96280700	96825966	rs754413	96530700	A	G	0.93 (0.36,2.38)	0.88	0.90 (0.72,1.13)	0.358	0.78 (0.69,0.87)	<0.001	0.28
11	26	96280700	96825966	rs3018644	96575966	G	A	1.56 (0.58,4.19)	0.376	0.90 (0.72,1.12)	0.352	0.77 (0.68,0.87)	<0.001	0.075
12	27	47212480	47712480	rs74523587	47462480	A	C	0.78 (0.70,0.87)	<0.001	1.16 (0.73,1.84)	0.541	-	-	0.15

12	28	55968568	56468568	rs73119275	56218568	C	T	0.78 (0.70,0.87)	<0.001	1.37 (0.83,2.27)	0.221	-	-	0.024
12	29	69525118	70042102	rs2870901	69775118	C	T	0.57 (0.41,0.78)	<0.001	0.70 (0.59,0.83)	<0.001	0.97 (0.84,1.13)	0.719	<0.001
12	29	69525118	70042102	rs1585705	69792102	A	C	0.53 (0.38,0.73)	<0.001	0.72 (0.61,0.85)	<0.001	0.97 (0.83,1.12)	0.665	<0.001
12	30	104063861	104572214	rs11111842	104313861	A	G	0.75 (0.66,0.84)	<0.001	1.20 (0.91,1.57)	0.192	4.86 (1.02,23.11)	0.047	<0.001
12	30	104063861	104572214	rs10861145	104314531	G	C	0.75 (0.66,0.84)	<0.001	1.20 (0.91,1.57)	0.193	4.86 (1.02,23.11)	0.047	<0.001
12	30	104063861	104572214	rs11833702	104314560	A	G	0.74 (0.66,0.83)	<0.001	1.21 (0.92,1.58)	0.176	4.86 (1.02,23.11)	0.047	<0.001
12	30	104063861	104572214	rs11111843	104314956	T	C	0.75 (0.66,0.84)	<0.001	1.20 (0.91,1.57)	0.193	4.86 (1.02,23.11)	0.047	<0.001
12	30	104063861	104572214	rs11111844	104316005	T	C	0.75 (0.66,0.84)	<0.001	1.20 (0.91,1.57)	0.191	4.86 (1.02,23.11)	0.047	<0.001
12	30	104063861	104572214	rs76025677	104317050	A	G	0.75 (0.66,0.84)	<0.001	1.21 (0.92,1.58)	0.174	4.86 (1.02,23.11)	0.047	<0.001
12	30	104063861	104572214	rs11111846	104318346	T	C	0.75 (0.66,0.84)	<0.001	1.20 (0.91,1.57)	0.191	4.86 (1.02,23.11)	0.047	<0.001
12	30	104063861	104572214	rs11111849	104319873	A	G	0.75 (0.66,0.84)	<0.001	1.20 (0.91,1.57)	0.192	4.86 (1.02,23.11)	0.047	<0.001
12	30	104063861	104572214	rs11111850	104321073	A	T	0.75 (0.66,0.84)	<0.001	1.20 (0.91,1.57)	0.191	4.86 (1.02,23.11)	0.047	<0.001
12	30	104063861	104572214	rs3794246	104321902	T	C	0.75 (0.67,0.84)	<0.001	1.20 (0.91,1.57)	0.194	4.87 (1.03,23.18)	0.046	<0.001
12	30	104063861	104572214	rs17034916	104322214	C	T	0.75 (0.66,0.84)	<0.001	1.20 (0.91,1.57)	0.191	4.86 (1.02,23.11)	0.047	<0.001
13	31	26140990	26728255	rs117674980	26390990	A	G	0.85 (0.76,0.95)	0.003	0.38 (0.24,0.60)	<0.001	-	-	<0.001
13	31	26140990	26728255	rs77999054	26394850	G	A	0.85 (0.76,0.95)	0.003	0.38 (0.24,0.60)	<0.001	-	-	<0.001
13	31	26140990	26728255	rs3783124	26458231	C	T	0.85 (0.76,0.95)	0.003	0.37 (0.23,0.59)	<0.001	-	-	<0.001
13	31	26140990	26728255	rs74335017	26478255	C	T	0.85 (0.76,0.94)	0.003	0.37 (0.23,0.59)	<0.001	-	-	<0.001
13	32	67331127	67831127	rs9571707	67581127	A	G	0.78 (0.67,0.90)	<0.001	0.83 (0.70,0.98)	0.028	0.82 (0.57,1.18)	0.289	0.603
13	33	93315090	93875433	rs1932193	93565090	A	C	0.78 (0.65,0.94)	0.008	0.81 (0.70,0.94)	0.007	0.83 (0.65,1.07)	0.15	0.714
13	33	93315090	93875433	rs306675	93606083	A	C	0.81 (0.63,1.03)	0.09	0.81 (0.70,0.95)	0.007	0.81 (0.67,0.98)	0.027	0.951
13	33	93315090	93875433	rs306677	93613362	C	G	0.81 (0.63,1.04)	0.092	0.82 (0.70,0.95)	0.008	0.81 (0.67,0.98)	0.028	0.94

13	33	93315090	93875433	rs306679	93625433	G	T	0.87 (0.67,1.12)	0.281	0.82 (0.70,0.95)	0.01	0.77 (0.64,0.93)	0.006	0.42
13	34	100252104	100752104	rs72653992	100502104	A	G	0.75 (0.67,0.84)	<0.001	1.54 (1.09,2.18)	0.014	1.40 (0.27,7.27)	0.692	<0.001
14	35	57055847	57555847	rs12896185	57305847	A	G	0.81 (0.71,0.93)	0.002	0.77 (0.64,0.93)	0.006	0.87 (0.54,1.43)	0.589	0.961
14	36	80321393	80823172	rs1181351	80571393	G	T	1.09 (0.77,1.55)	0.614	0.97 (0.82,1.14)	0.687	0.65 (0.56,0.76)	<0.001	<0.001
14	36	80321393	80823172	rs28444185	80573172	A	T	0.65 (0.56,0.76)	<0.001	0.97 (0.82,1.13)	0.665	1.10 (0.78,1.55)	0.571	<0.001
15	37	90077226	90587639	rs28698386	90327226	T	C	0.88 (0.71,1.08)	0.22	0.78 (0.67,0.91)	0.001	0.81 (0.66,1.01)	0.059	0.621
15	37	90077226	90587639	rs6496605	90337639	C	T	0.87 (0.71,1.07)	0.194	0.78 (0.67,0.90)	<0.001	0.78 (0.63,0.96)	0.019	0.485
16	38	13669429	14169429	rs75422462	13919429	G	A	0.79 (0.71,0.88)	<0.001	1.09 (0.64,1.84)	0.752	-	-	0.292
18	39	52608233	53108233	rs34352315	52858233	G	A	0.82 (0.73,0.91)	<0.001	0.42 (0.21,0.84)	0.014	-	-	0.043
21	40	17158456	17658456	rs2823575	17408456	G	A	0.70 (0.62,0.80)	<0.001	1.24 (1.00,1.54)	0.053	0.92 (0.39,2.18)	0.85	<0.001
21	41	40203038	40703038	rs74482819	40453038	C	T	0.76 (0.68,0.85)	<0.001	2.15 (1.34,3.45)	0.002	-	-	<0.001
22	42	18315772	18842083	rs71328255	18565772	A	G	0.80 (0.72,0.89)	<0.001	1.04 (0.60,1.81)	0.876	-	-	0.501
22	42	18315772	18842083	rs71328256	18569926	T	C	0.80 (0.72,0.89)	<0.001	1.04 (0.60,1.81)	0.879	-	-	0.503
22	42	18315772	18842083	rs34680388	18576176	T	A	0.80 (0.72,0.89)	<0.001	1.07 (0.61,1.86)	0.818	-	-	0.441
22	42	18315772	18842083	rs13058445	18580087	A	G	0.80 (0.72,0.89)	<0.001	1.04 (0.60,1.80)	0.884	-	-	0.508
22	42	18315772	18842083	rs34658760	18584324	A	C	0.80 (0.72,0.89)	<0.001	1.07 (0.62,1.86)	0.812	-	-	0.441
22	42	18315772	18842083	rs34667409	18584379	A	G	0.80 (0.72,0.89)	<0.001	1.04 (0.60,1.81)	0.882	-	-	0.506
22	42	18315772	18842083	rs35137695	18587898	C	T	0.80 (0.72,0.89)	<0.001	1.07 (0.61,1.85)	0.819	-	-	0.446
22	42	18315772	18842083	rs13058179	18592083	G	A	0.79 (0.71,0.88)	<0.001	1.05 (0.61,1.82)	0.861	-	-	0.483
22	43	29569106	30069106	rs144548824	29819106	G	A	0.81 (0.73,0.90)	<0.001	0.73 (0.47,1.15)	0.18	-	-	0.606

[1]The risk of fish oil supplementation for the development of vascular dementia was evaluated using Cox regression models adjusted by age, sex, and top 10 genetic principal components within each SNP genotype. Genotype groups with five or fewer incident cases were not considered in the subgroup analysis.

Abbreviations: ALT, alternative allele; CHR, chromosome; CI, confidence interval; HR, hazard ratio; POS, SNP position with GRCh37 assembly; P-interaction, *p*-value of the interaction terms; REF, reference allele; SNP, single nucleotide polymorphism.

Table S8. 43 Loci interacting with FOS in the development of dementia compared to known GWAS loci.

CHR	No. Loci	Start	End	VEP gene[1]	Closest gene[2]	Distance (bp)	Known loci[3]
1	1	8027198	8527198	-	*SLC45A1*	-100,688	-
1	2	14816263	15316263	*KAZN*	*KAZN*	0	-
1	3	29232654	29732654	*SRSF4*	*SRSF4*	0	Omega-3% (Karjalainen et al, 2024)
1	4	101852308	102352308	-	*OLFM3*	-360,278	-
1	5	106483537	106983795	-	*PRMT6*	-865,472	-
1	6	110597781	111098352	-	*RBM15*	-32,776	-
1	7	222375645	222876659	-	*HHIPL2*	-94,786	-
2	8	100571438	101071438	-	*AFF3*	+62,237	-
2	9	170757664	171257664	-	*MYO3B*	-26,991	-
4	10	16312945	16812945	*LDB2*	*LDB2*	0	-
4	11	68145282	68645282	*CENPC*	*CENPC*	0	-
4	12	114132769	114635835	*CAMK2D*	*CAMK2D*	0	-
5	13	3009680	3517512	-	*IRX1*	-333,444	-
5	14	100147117	100647117	-	*ST8SIA4*	+158,147	-
6	15	767009	1267009	-	*AL033381.1*	-63,155	-
6	16	115069829	115569829	-	*HS3ST5*	+655,620	-
6	17	166801161	167302048	*RPS6KA2*	*RPS6KA2*	0	-
7	18	20096169	20596169	-	*ITGB8*	-24,156	-
7	19	143574310	144074310	*OR2A14*	*OR2A14*	-1,841	-
8	20	3675912	4175912	*CSMD1*	*CSMD1*	0	-
8	21	82617116	83117116	-	*SNX16*	+112,015	-
8	22	111221071	111721071	-	*KCNV1*	+482,995	-
10	23	4149849	4649849	-	*AKR1E2*	-428,972	-
10	24	129615006	130115006	*PTPRE*	*PTPRE*	0	-
11	25	85731587	86233703	*EED*	*EED*	0	ADRD (Bellenguez et al., 2022)
11	26	96280700	96825966	-	*JRKL*	+452,813	-
12	27	47212480	47712480	-	*PCED1B*	-10,906	-
12	28	55968568	56468568	*DNAJC14, ORMDL2*	*RP11-762I7.5; DNAJC14*	0	-
12	29	69525118	70042102	*YEATS4*	*YEATS4*	+38,619	-
12	30	104063861	104572214	*HSP90B1*	*HSP90B1*	-9,325	-
13	31	26140990	26728255	*ATP8A2, AL138815.1*	*ATP8A2*	0	-
13	32	67331127	67831127	*PCDH9*	*PCDH9*	0	-
13	33	93315090	93875433	-	*GPC6*	-273,012	-
13	34	100252104	100752104	*CLYBL*	*CLYBL*	0	-
14	35	57055847	57555847	-	*OTX2*	+28,650	-
14	36	80321393	80823172	-	*DIO2*	-280,928	-
15	37	90077226	90587639	*ANPEP*	*MESP2*	+23,404	-

16	38	13669429	14169429	-	*ERCC4*	-94,585	-
18	39	52608233	53108233	-	*CCDC68*	+231,494	-
21	40	17158456	17658456	-	*USP25*	+306,112	-
21	41	40203038	40703038	-	*PSMG1*	-102,739	Omega-3 PUFAs (Richardson et al, 2022; Francis et al, 2022; Borges et al, 2022; Davyson et al, 2023) Omega-6/omega-3 (Richardson et al, 2022; Borges et al, 2022)
22	42	18315772	18842083	*PEX26, TUBA8*	*PEX26*	0	-
22	43	29569106	30069106	*AP1B1*	*AP1B1*	0	-

[1]Gene symbols were annotated by the VEP website, indicating which SNPs affected their corresponding protein-coding transcripts.

[2]Closest genes were identified as the protein-coding genes with the shortest physical distance to the top interaction signal at each locus, based on the GRCh37 genome build from the Ensembl database, and the distance was defined by the position of the top interaction signal and the start codon site of the protein-coding gene.

[3]Comparison between interaction loci and reported loci related to Alzheimer's disease and related dementias and polyunsaturated fatty acids (PUFA) traits [1-6]. No time-to-event GWAS locus of all-cause dementia, Alzheimer's disease, and vascular dementia overlapped with these 43 interaction loci.

Abbreviations: ADRD, Alzheimer's disease and related dementia; bp, base pair; CHR, chromosome; GWAS, genome-wide association study; PUFA, polyunsaturated fatty acid; VEP, Variant Effect Predictor.

Table S9. Association between interaction loci and dementia outcomes in the whole dataset and FOS subgroups.

CHR	No. Loci	Top SNP[1]	P-interaction	All[2]		Fish oil users[2]		Non-fish oil users[2]		Patterns of FOS effects[3]
				HR (95%CI)	*p*-value	HR (95%CI)	*p*-value	HR (95%CI)	*p*-value	
All-cause dementia										
1	2	rs75837905	3.70E-05	1.02 (0.92,1.14)	6.99E-01	1.34 (1.14,1.57)	3.17E-04	0.84 (0.72,0.97)	2.20E-02	OP
1	3	rs116264291	1.78E-04	1.16 (1.03,1.31)	1.81E-02	1.51 (1.27,1.81)	**4.47E-06**	0.95 (0.80,1.13)	5.38E-01	OP
1	4	rs61804494	1.45E-04	0.94 (0.90,0.99)	1.42E-02	0.84 (0.77,0.91)	**9.08E-06**	1.01 (0.95,1.07)	7.36E-01	N
1	5	rs12043527	8.37E-05	1.00 (0.94,1.06)	9.51E-01	1.15 (1.05,1.25)	2.01E-03	0.91 (0.85,0.98)	1.52E-02	N
1	6	rs12024264	1.52E-06	1.05 (0.97,1.14)	2.16E-01	1.32 (1.17,1.48)	**4.05E-06**	0.89 (0.80,1.00)	4.25E-02	OP
2	9	rs4435418	2.71E-04	1.05 (1.02,1.09)	2.91E-03	1.14 (1.08,1.21)	**2.47E-06**	1.00 (0.96,1.05)	8.99E-01	N
6	16	rs9505681	4.10E-05	1.16 (1.01,1.34)	4.02E-02	1.64 (1.34,2.01)	**2.18E-06**	0.90 (0.73,1.10)	3.00E-01	OP
6	17	rs963302	3.98E-06	0.97 (0.93,1.01)	1.05E-01	0.86 (0.81,0.92)	**3.36E-06**	1.04 (0.99,1.09)	1.22E-01	N
7	18	rs3114430	2.09E-05	0.97 (0.94,1.01)	9.17E-02	0.88 (0.83,0.93)	**8.95E-06**	1.03 (0.98,1.08)	1.92E-01	N
7	19	rs12670543	3.82E-06	1.04 (0.98,1.10)	1.87E-01	1.22 (1.12,1.33)	**6.15E-06**	0.93 (0.86,1.00)	6.54E-02	OP
10	24	rs4750683	2.44E-04	0.94 (0.90,0.98)	1.24E-03	0.86 (0.80,0.91)	**8.99E-07**	0.99 (0.95,1.04)	8.12E-01	N
11	26	rs3018644	6.01E-05	1.07 (1.02,1.13)	9.25E-03	0.94 (0.87,1.02)	1.27E-01	1.16 (1.09,1.24)	**7.62E-06**	N
12	27	rs74523587	1.54E-04	1.16 (1.04,1.30)	1.05E-02	1.49 (1.26,1.76)	**2.41E-06**	0.97 (0.83,1.13)	6.71E-01	OP
12	28	rs73119275	6.15E-06	1.10 (0.97,1.24)	1.46E-01	1.52 (1.27,1.81)	**3.42E-06**	0.86 (0.72,1.02)	8.18E-02	OP
14	35	rs12896185	5.71E-05	1.04 (1.00,1.08)	5.07E-02	1.15 (1.08,1.23)	**9.16E-06**	0.97 (0.93,1.03)	3.16E-01	OP
22	42	rs35137695	6.68E-06	1.18 (1.04,1.34)	1.31E-02	1.64 (1.37,1.96)	**6.90E-08**	0.90 (0.74,1.08)	2.60E-01	N
22	43	rs144548824	1.83E-04	1.15 (1.04,1.28)	7.85E-03	0.86 (0.71,1.05)	1.30E-01	1.33 (1.18,1.51)	**4.89E-06**	N
Alzheimer's disease										
1	1	rs116501531	5.30E-05	1.28 (1.06,1.55)	1.03E-02	1.92 (1.50,2.46)	**2.42E-07**	0.87 (0.65,1.16)	3.41E-01	P
1	2	rs75837905	2.15E-05	1.17 (1.00,1.37)	4.80E-02	1.68 (1.36,2.06)	**1.20E-06**	0.84 (0.66,1.06)	1.42E-01	P
1	6	rs12024264	2.50E-05	1.09 (0.97,1.23)	1.44E-01	1.43 (1.21,1.68)	1.81E-05	0.87 (0.73,1.03)	9.90E-02	P
4	11	rs149325653	1.31E-04	1.28 (1.04,1.56)	1.69E-02	0.65 (0.42,1.00)	5.22E-02	1.70 (1.36,2.13)	**3.29E-06**	N
5	13	rs59781823	5.87E-05	1.08 (1.02,1.13)	7.62E-03	0.94 (0.86,1.03)	1.75E-01	1.17 (1.10,1.26)	**4.27E-06**	OP
8	20	rs2042528	7.34E-05	1.07 (1.02,1.12)	1.10E-02	0.94 (0.87,1.02)	1.47E-01	1.16 (1.09,1.24)	**7.04E-06**	OP
8	22	rs116871946	2.06E-04	1.18 (1.05,1.32)	4.57E-03	0.88 (0.72,1.08)	2.09E-01	1.38 (1.21,1.59)	**2.53E-06**	N
11	25	rs78851816	5.75E-06	1.12 (1.02,1.23)	1.32E-02	1.42 (1.24,1.62)	**1.71E-07**	0.93 (0.82,1.06)	2.81E-01	P
13	32	rs9571707	1.83E-06	1.04 (0.98,1.09)	2.11E-01	1.21 (1.12,1.32)	**7.39E-06**	0.93 (0.86,1.00)	3.82E-02	OP
13	33	rs306675	1.95E-05	1.06 (1.00,1.11)	4.01E-02	1.22 (1.12,1.32)	**2.96E-06**	0.96 (0.90,1.03)	2.35E-01	OP

15	37	rs28698386	1.20E-04	0.94 (0.89,0.99)	1.72E-02	0.83 (0.77,0.90)	**5.96E-06**	1.02 (0.96,1.09)	5.26E-01	P
16	38	rs75422462	2.28E-04	1.26 (1.05,1.50)	1.17E-02	1.78 (1.40,2.26)	**1.98E-06**	0.91 (0.69,1.19)	4.94E-01	P
18	39	rs34352315	3.42E-05	1.36 (1.11,1.66)	2.87E-03	0.63 (0.40,1.00)	4.86E-02	1.88 (1.50,2.35)	**4.44E-08**	N
22	43	rs144548824	2.06E-04	1.09 (0.93,1.28)	2.81E-01	0.69 (0.50,0.94)	2.02E-02	1.37 (1.14,1.65)	8.93E-04	N
Vascular dementia										
1	5	rs11184799	5.86E-05	1.15 (1.04,1.27)	7.08E-03	1.49 (1.27,1.74)	**6.05E-07**	0.98 (0.86,1.11)	7.49E-01	N
1	7	rs17163137	3.82E-05	0.96 (0.89,1.03)	2.35E-01	0.77 (0.68,0.88)	6.29E-05	1.07 (0.98,1.17)	1.44E-01	N
2	8	rs113777826	1.20E-04	1.37 (1.05,1.78)	1.83E-02	2.39 (1.70,3.37)	**5.37E-07**	0.84 (0.56,1.27)	4.06E-01	OP
4	10	rs148811174	6.38E-08	1.07 (0.98,1.17)	1.16E-01	0.73 (0.62,0.87)	3.35E-04	1.27 (1.14,1.41)	**6.37E-06**	N
4	12	rs13106836	5.65E-06	1.06 (0.99,1.14)	1.19E-01	1.33 (1.18,1.50)	**3.58E-06**	0.94 (0.85,1.02)	1.45E-01	OP
5	14	rs2089903	7.41E-07	0.86 (0.68,1.08)	1.84E-01	0.46 (0.34,0.61)	**1.44E-07**	1.52 (1.05,2.21)	2.67E-02	OP
6	15	rs845890	1.49E-04	1.13 (1.04,1.23)	5.29E-03	1.40 (1.22,1.61)	**1.81E-06**	1.00 (0.89,1.11)	9.56E-01	N
8	21	rs117216345	2.14E-05	1.14 (1.00,1.29)	4.75E-02	1.59 (1.32,1.93)	**1.67E-06**	0.91 (0.76,1.08)	2.82E-01	N
10	23	rs116876958	1.65E-04	1.33 (1.10,1.62)	3.05E-03	2.03 (1.55,2.65)	**2.28E-07**	0.98 (0.74,1.29)	8.63E-01	OP
12	29	rs1585705	6.05E-05	0.91 (0.84,0.98)	1.26E-02	1.13 (0.99,1.29)	6.60E-02	0.81 (0.74,0.89)	**8.51E-06**	N
12	30	rs11833702	1.96E-04	1.19 (1.04,1.36)	1.24E-02	1.63 (1.33,1.99)	**3.04E-06**	0.97 (0.81,1.16)	7.44E-01	OP
13	31	rs3783124	2.08E-04	1.28 (1.06,1.53)	8.79E-03	0.67 (0.44,1.02)	5.91E-02	1.61 (1.32,1.98)	**4.15E-06**	N
13	34	rs72653992	5.78E-05	1.37 (1.16,1.62)	2.26E-04	2.06 (1.62,2.61)	**2.88E-09**	1.02 (0.80,1.29)	8.76E-01	OP
14	36	rs28444185	8.16E-05	1.09 (1.01,1.18)	2.59E-02	1.34 (1.18,1.52)	**5.21E-06**	0.97 (0.88,1.07)	5.56E-01	N
21	40	rs2823575	4.40E-05	1.11 (1.00,1.24)	5.99E-02	1.47 (1.24,1.73)	**6.02E-06**	0.93 (0.80,1.07)	3.16E-01	N
21	41	rs74482819	1.74E-05	1.25 (0.99,1.58)	6.56E-02	2.18 (1.61,2.97)	**6.10E-07**	0.76 (0.53,1.10)	1.46E-01	OP

[1]The most significant interaction SNPs in loci for each outcome.

[2]The association between interaction signals and dementia outcomes was evaluated by Cox regression models adjusted by age, sex, and top 10 genetic principal components.

[3]Patterns of fish oil supplement effects on dementia outcomes across genotype subgroups. N, indicated "only negative", meaning fish oil supplements showed consistent negative association with incident outcomes across all significant genotype subgroups ($p < 0.05$); P, indicated "only positive", meaning fish oil supplements showed consistent positive association with incident outcomes across all significant genotype subgroups ($p < 0.05$); OP, indicated "opposite", meaning fish oil supplements showed opposite directions of association with incident outcomes across all significant genotype subgroups ($p < 0.05$).

Abbreviations: CHR, chromosome; CI, confidence interval; FOS, fish oil supplements; HR, hazard ratio; POS, SNP position with GRCh37 assembly; P-interaction, *p*-value of the interaction terms; SNP, single nucleotide polymorphism.

Table S10. Replicated loci from interaction analyses of FOS status from 24-hour recall questionnaire, oily fish intake, and circulating omega-3 levels.

No. Loci	CHR	SNP	POS	REF	ALT	Main (N=357,631)		24-h recall FOS (N=156,004)		Oily fish intake (N=356,052)		Omega-3 (N=200,980)	
						Beta	*p*-value	Beta	*p*-value	Beta	*p*-value	Beta	*p*-value
All-cause dementia													
Loci_FOinteract_4	1	rs61804494	102102308	G	A	**-0.188**	**<0.001**	-0.081	0.407	-0.014	0.778	**-0.438**	**0.003**
Loci_FOinteract_6	1	rs12024138	110847781	C	T	**0.391**	**<0.001**	0.258	0.121	-0.014	0.87	**0.47**	**0.049**
Loci_FOinteract_6	1	rs12024264	110848352	C	A	**0.392**	**<0.001**	0.258	0.12	-0.014	0.865	**0.473**	**0.048**
Loci_FOinteract_17	6	rs6941412	167051161	C	T	**-0.182**	**<0.001**	-0.119	0.129	-0.052	0.192	**-0.235**	**0.05**
Loci_FOinteract_17	6	rs963302	167052048	C	G	**-0.184**	**<0.001**	-0.149	0.056	-0.053	0.183	**-0.235**	**0.047**
Loci_FOinteract_18	7	rs3114430	20346169	C	A	**-0.16**	**<0.001**	**-0.157**	**0.032**	0.072	0.058	0.003	0.981
Loci_FOinteract_19	7	rs12670543	143824310	C	A	**0.27**	**<0.001**	0.074	0.527	**0.182**	**0.003**	0.157	0.377
Loci_FOinteract_26	11	rs3018644	96575966	G	A	**-0.213**	**<0.001**	**-0.212**	**0.043**	-0.057	0.297	0.18	0.271
Loci_FOinteract_42	22	rs71328255	18565772	A	G	**0.586**	**<0.001**	**0.49**	**0.037**	-0.032	0.819	0.616	0.116
Loci_FOinteract_42	22	rs71328256	18569926	T	C	**0.584**	**<0.001**	**0.491**	**0.037**	-0.032	0.818	0.619	0.114
Loci_FOinteract_42	22	rs34680388	18576176	T	A	**0.574**	**<0.001**	**0.494**	**0.036**	-0.024	0.863	0.658	0.09
Loci_FOinteract_42	22	rs13058445	18580087	A	G	**0.581**	**<0.001**	**0.491**	**0.037**	-0.031	0.824	0.622	0.113
Loci_FOinteract_42	22	rs34658760	18584324	A	C	**0.591**	**<0.001**	**0.515**	**0.03**	-0.039	0.777	0.623	0.112
Loci_FOinteract_42	22	rs34667409	18584379	A	G	**0.582**	**<0.001**	**0.491**	**0.037**	-0.032	0.816	0.623	0.112
Loci_FOinteract_42	22	rs35137695	18587898	C	T	**0.597**	**<0.001**	**0.515**	**0.03**	-0.033	0.811	0.668	0.086
Loci_FOinteract_42	22	rs13058179	18592083	G	A	**0.562**	**<0.001**	**0.494**	**0.036**	-0.044	0.748	0.56	0.16
Loci_FOinteract_43	22	rs144548824	29819106	G	A	**-0.44**	**<0.001**	-0.34	0.133	**-0.329**	**0.002**	0.105	0.745
Alzheimer's disease													
Loci_FOinteract_1	1	rs116501531	8277198	A	G	**0.793**	**<0.001**	**0.863**	**0.017**	0.428	0.052	0.285	0.598
Loci_FOinteract_25	11	rs78851816	85983703	G	A	**0.422**	**<0.001**	**0.384**	**0.039**	-0.046	0.632	**0.561**	**0.039**
Loci_FOinteract_32	13	rs9571707	67581127	A	G	**0.272**	**<0.001**	0.166	0.135	**0.137**	**0.021**	0.208	0.228
Loci_FOinteract_33	13	rs1932193	93565090	A	C	**0.197**	**<0.001**	-0.014	0.895	0.007	0.897	**0.325**	**0.039**

Loci_FOinteract_38	16	rs75422462	13919429	G	A	**0.678**	**<0.001**	0.285	0.408	0.197	0.319	**1.657**	**<0.001**
Vascular dementia													
Loci_FOinteract_12	4	rs13123494	114382769	C	A	**0.347**	**<0.001**	**0.484**	**0.004**	0.015	0.846	**0.456**	**0.029**
Loci_FOinteract_12	4	rs1525000	114384869	C	T	**0.335**	**<0.001**	**0.468**	**0.006**	0.013	0.869	**0.478**	**0.022**
Loci_FOinteract_12	4	rs62314976	114385676	T	C	**0.344**	**<0.001**	**0.448**	**0.008**	0.019	0.803	**0.507**	**0.015**
Loci_FOinteract_12	4	rs13106836	114385835	G	A	**0.347**	**<0.001**	**0.467**	**0.006**	0.016	0.839	**0.499**	**0.017**
Loci_FOinteract_15	6	rs845890	1017009	C	A	**0.341**	**<0.001**	**0.572**	**0.004**	0.028	0.763	0.074	0.771
Loci_FOinteract_29	12	rs2870901	69775118	C	T	**0.321**	**<0.001**	**0.397**	**0.028**	0.058	0.465	0.063	0.782
Loci_FOinteract_31	13	rs117674980	26390990	A	G	**-0.86**	**<0.001**	**-2.441**	**0.017**	0.142	0.466	**-1.22**	**0.048**
Loci_FOinteract_31	13	rs77999054	26394850	G	A	**-0.863**	**<0.001**	**-2.445**	**0.017**	0.142	0.468	**-1.219**	**0.049**
Loci_FOinteract_31	13	rs3783124	26458231	C	T	**-0.887**	**<0.001**	**-2.426**	**0.018**	0.087	0.656	-1.125	0.07
Loci_FOinteract_31	13	rs74335017	26478255	C	T	**-0.885**	**<0.001**	**-2.384**	**0.02**	0.061	0.754	-1.108	0.076
Loci_FOinteract_36	14	rs1181351	80571393	G	T	**-0.322**	**<0.001**	-0.025	0.894	**-0.165**	**0.05**	**-0.649**	**0.004**
Loci_FOinteract_36	14	rs28444185	80573172	A	T	**0.322**	**<0.001**	0.029	0.874	0.16	0.056	**0.622**	**0.005**
Loci_FOinteract_40	21	rs2823575	17408456	G	A	**0.458**	**<0.001**	**0.553**	**0.025**	0.016	0.888	-0.234	0.481
Loci_FOinteract_41	21	rs74482819	40453038	C	T	**1.048**	**<0.001**	0.623	0.19	**0.608**	**0.031**	**1.257**	**0.037**

This table demonstrated replicated loci that significantly interacted with exposures, including FOS status defined by a 24-hour recall questionnaire (Yes/No), oily fish intake (lower/higher), and circulating omega-3 levels, in relation to each type of dementia among the 178 significant SNPs from 43 loci. Betas and *p*-values for the SNP (coded by the number of REF alleles carried) × Exposure interaction terms were estimated using Cox regression models adjusted for age, sex, and the top 10 genetic principal components (Model 1). Interaction signals that were replicated ($p < 0.05$) are highlighted in bold.

Abbreviations: ALT, alternative allele; CHR, chromosome; FOS, fish oil supplements; POS, SNP position with GRCh37 assembly; REF, reference allele; SNP, single nucleotide polymorphism.

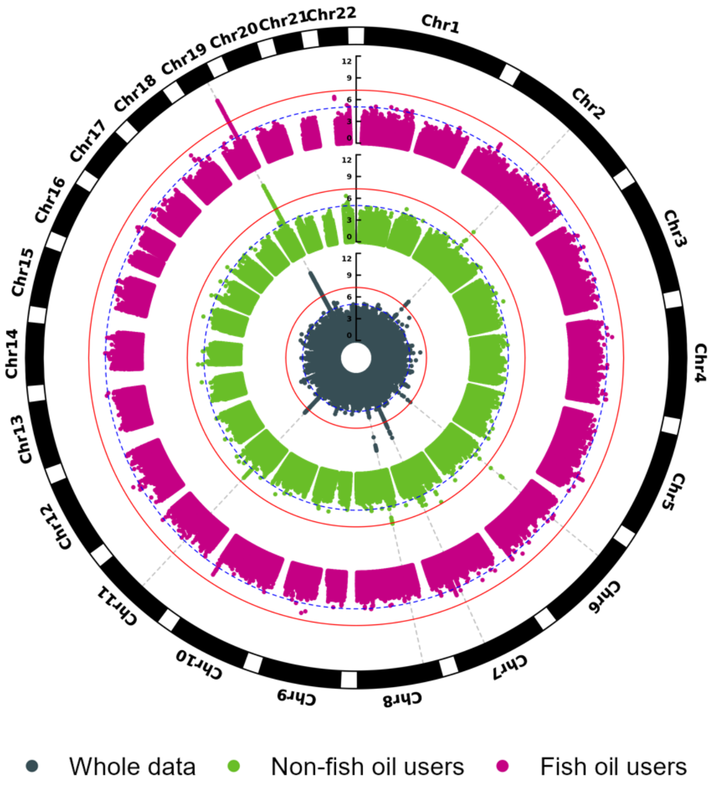

Figure S1. Manhattan plots of GWAS of all-cause dementia by FOS status.

Circular Manhattan plot for time-to-event GWAS of all-cause dementia in whole data (dark cyan), non-fish oil users (light green), and fish oil users (purple). Variants with a -log10(p) below 11 are not shown. Thresholds of genome-wide significance ($p < 5 \times 10^{-8}$) and suggestive significance ($p < 1 \times 10^{-5}$) were shown by a solid red line and dashed blue line, respectively. The positions of genome-wide significant signals were demonstrated by cross-circle grey dashed lines. Abbreviations: Chr, chromosome; FOS, fish oil supplements; GWAS, genome-wide association study.

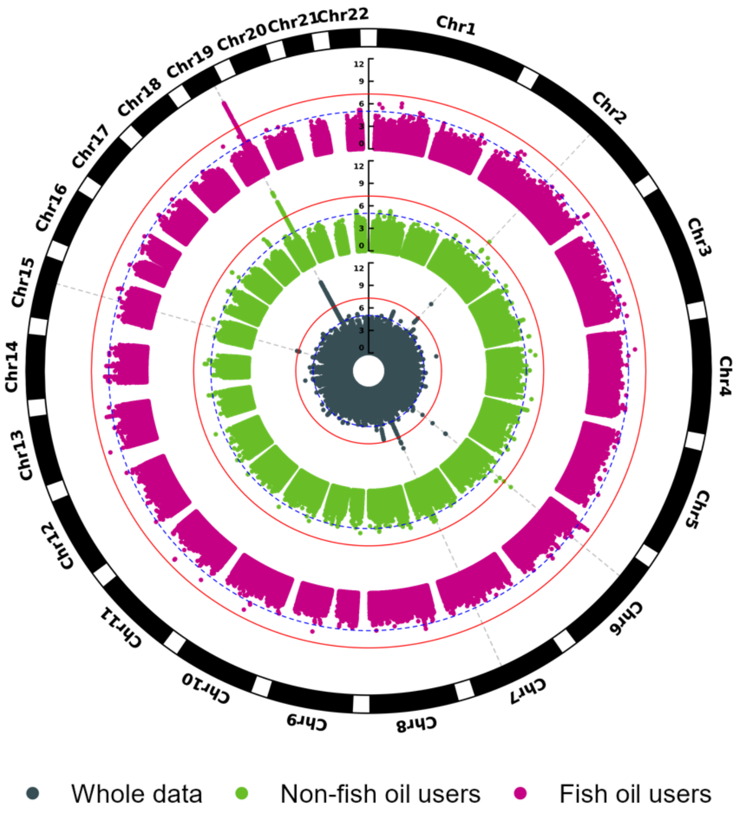

Figure S2. Manhattan plots of GWAS of Alzheimer's disease by FOS status.

Circular Manhattan plot for time-to-event GWAS of Alzheimer's disease in whole data (dark cyan), non-fish oil users (light green), and fish oil users (purple). Variants with a -log10(p) below 11 are not shown. Thresholds of genome-wide significance ($p < 5 \times 10^{-8}$) and suggestive significance ($p < 1 \times 10^{-5}$) were shown by a solid red line and dashed blue line, respectively. The positions of genome-wide significant signals were demonstrated by cross-circle grey dashed lines. Abbreviations: Chr, chromosome; FOS, fish oil supplements; GWAS, genome-wide association study.

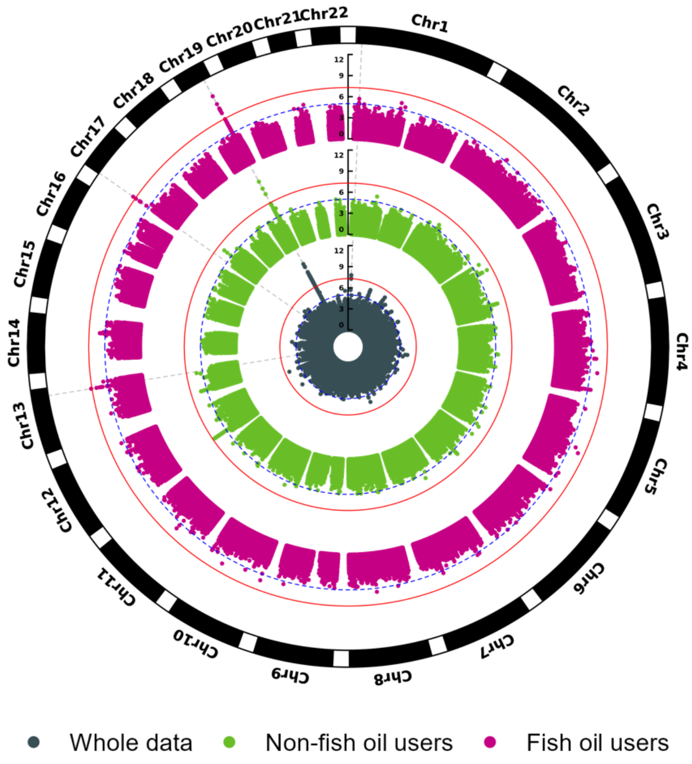

Figure S3. Manhattan plots of GWAS of vascular dementia by FOS status.

Circular Manhattan plot for time-to-event GWAS of vascular dementia in whole data (dark cyan), non-fish oil users (light green), and fish oil users (purple). Variants with a -log10(p) below 11 are not shown. Thresholds of genome-wide significance ($p < 5 \times 10^{-8}$) and suggestive significance ($p < 1 \times 10^{-5}$) were shown by a solid red line and dashed blue line, respectively. The positions of genome-wide significant signals were demonstrated by cross-circle grey dashed lines. Abbreviations: Chr, chromosome; FOS, fish oil supplements; GWAS, genome-wide association study.

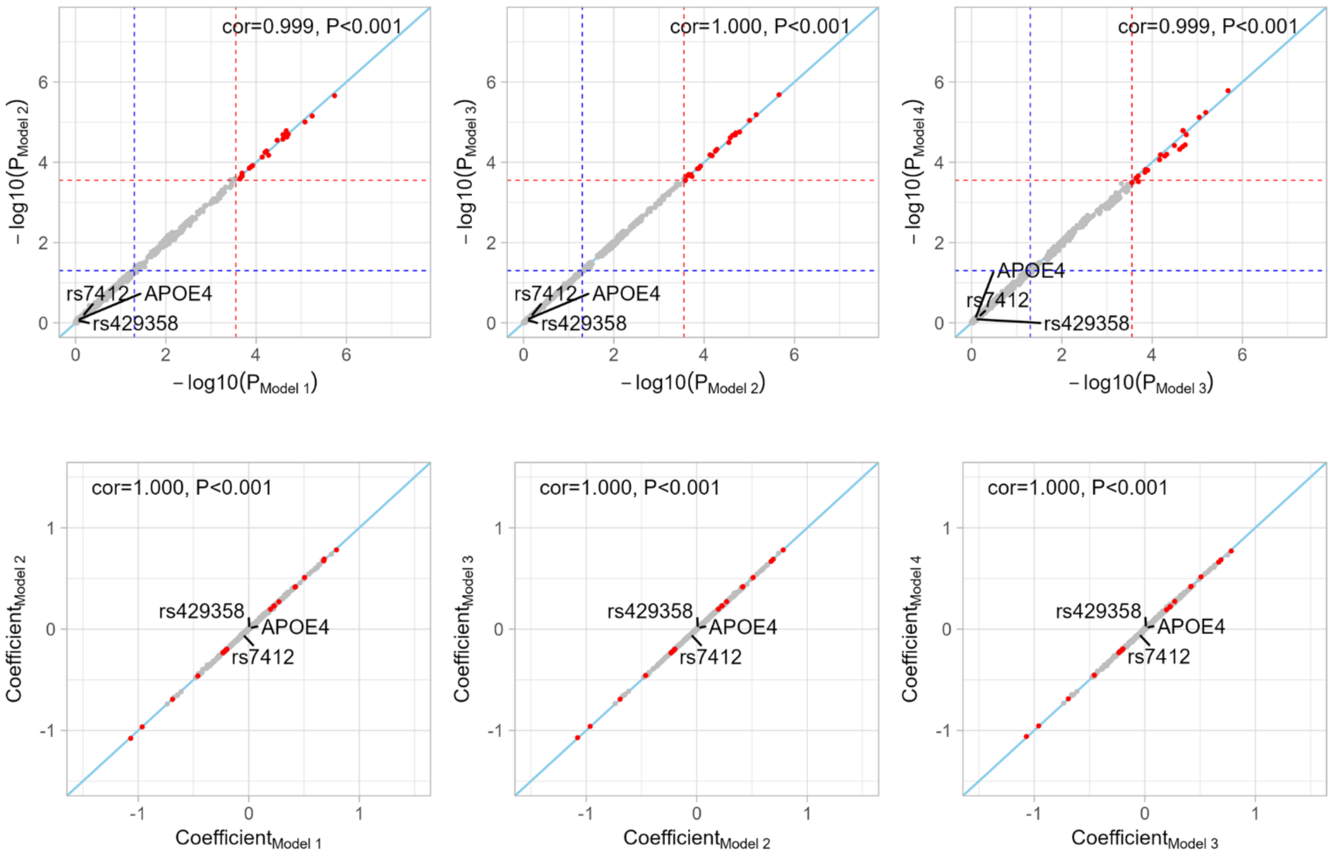

Figure S4. Comparison of results from interaction analysis of candidate SNPs with FOS in Alzheimer's disease using Model 1-4.

Model 1 was adjusted for basic covariates, including age, sex, top 10 genetic principal components, along with fish oil supplementation and the corresponding SNP. Model 2 was further adjusted for education, Townsend deprivation index (TDI), body mass index (BMI), smoking, alcohol intake, and physical activity. Model 3 additionally included 8 dietary patterns: oily fish intake, fruit intake, vegetable intake, processed meat intake, red meat intake, vitamin supplementation, mineral supplementation, and Glucosamine supplementation. Model 4 included 5 self-reported medical histories, hypertension, cardiovascular disease (CVD), high cholesterol, diabetes, and depression. The SNPs that significantly interacted with FOS status in Model 1 were shown as red points. The red dashed line and blue dashed line referred to the Bonferroni correction threshold ($p = 2.8 \times 10^{-4}$) and nominal significance threshold ($p = 0.05$), respectively. Pearson tests were conducted to assess the correlation of *p*-values or beta coefficients between models.
Abbreviations: cor, Pearson correlation coefficient; P, *p*-value of Pearson correlation test.

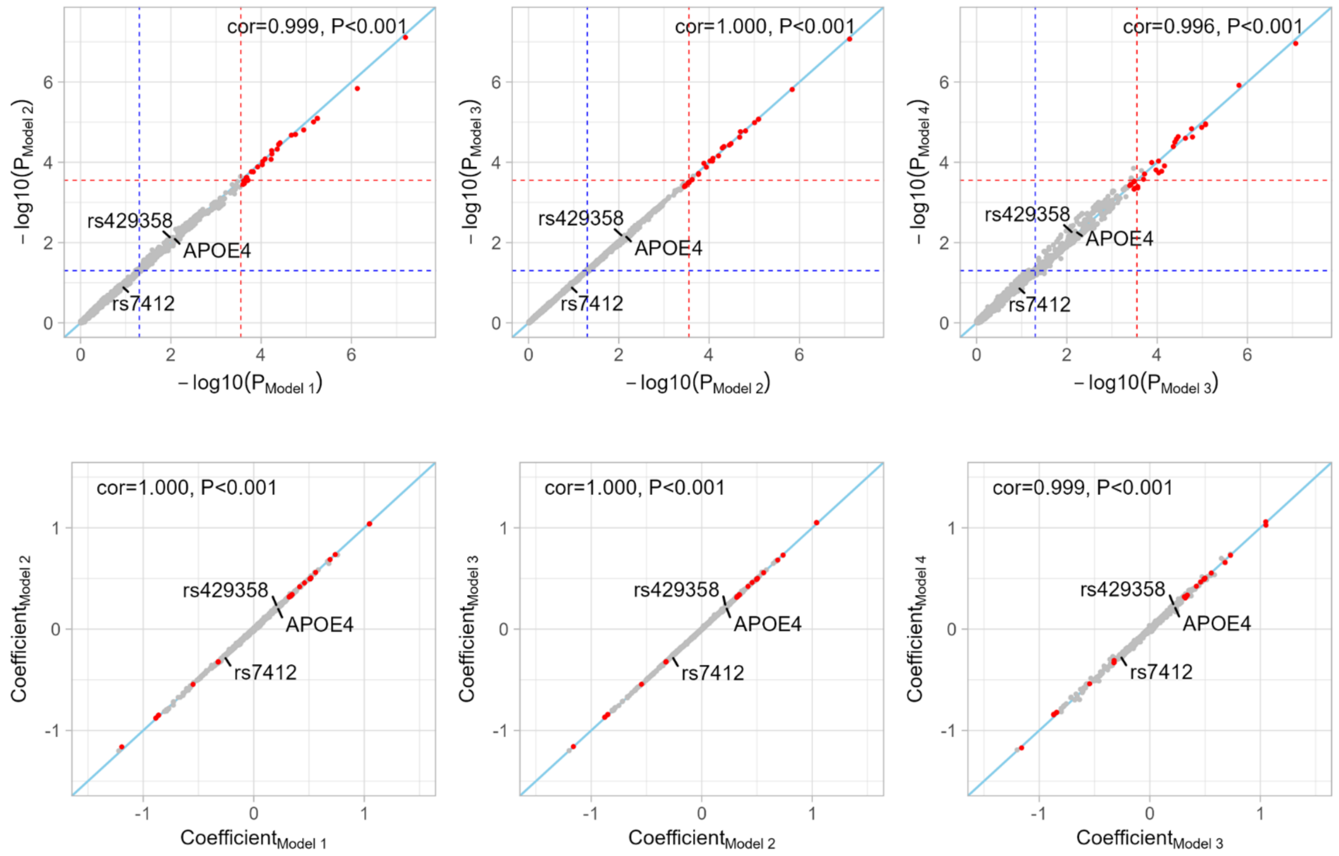

Figure S5. Comparison of results from interaction analysis of candidate SNPs with FOS in vascular dementia using Model 1-4.

Model 1 was adjusted for basic covariates, including age, sex, top 10 genetic principal components, along with fish oil supplementation and the corresponding SNP. Model 2 was further adjusted for education, Townsend deprivation index (TDI), body mass index (BMI), smoking, alcohol intake, and physical activity. Model 3 additionally included 8 dietary patterns: oily fish intake, fruit intake, vegetable intake, processed meat intake, red meat intake, vitamin supplementation, mineral supplementation, and Glucosamine supplementation. Model 4 included 5 self-reported medical histories, hypertension, cardiovascular disease (CVD), high cholesterol, diabetes, and depression. The SNPs that significantly interacted with FOS status in Model 1 were shown as red points. The red dashed line and blue dashed line referred to the Bonferroni correction threshold ($p = 2.8 \times 10^{-4}$) and nominal significance threshold ($p = 0.05$), respectively. Pearson tests were conducted to assess the correlation of *p*-values or beta coefficients between models.

Abbreviations: cor, Pearson correlation coefficient; P, *p*-value of Pearson correlation test.

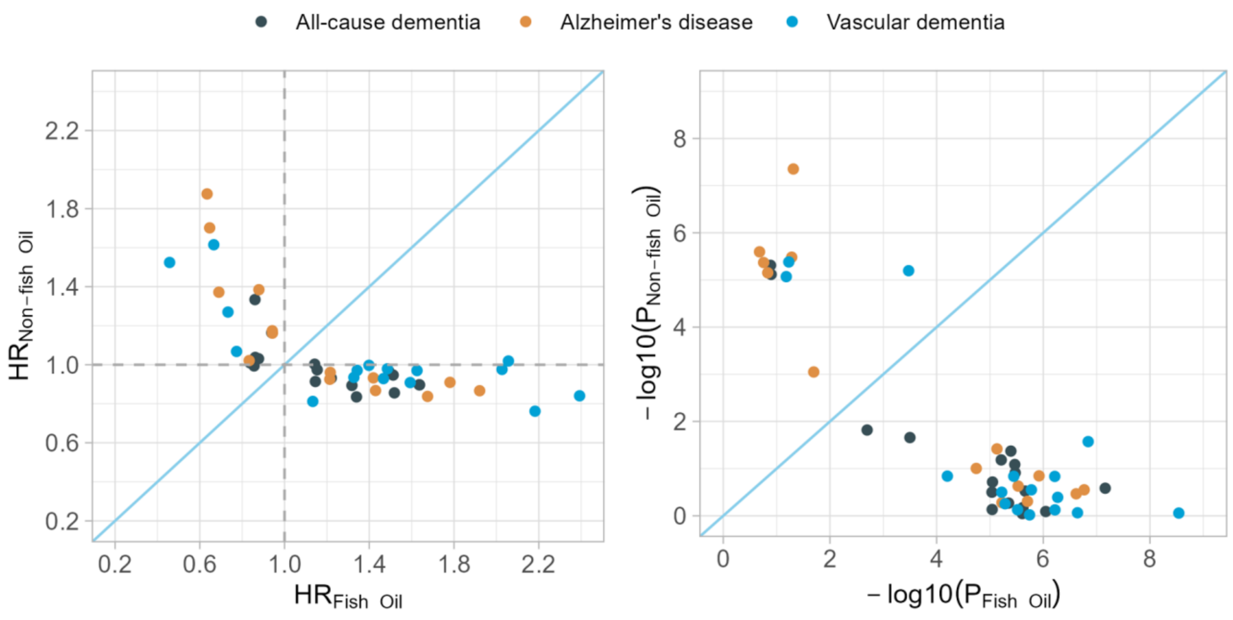

Figure S6. Comparison of the associations between interaction loci and dementia outcomes stratified by FOS status.

Each point was the top SNPs of interaction loci for all-cause dementia (dark cyan), Alzheimer's disease (orange), and vascular dementia (blue). The grey dashed lines represented no significant association between SNPs and incident dementia in the subgroups of fish oil users or non-users. The blue diagonal lines represented that the HR or *p*-values were equal in two subgroups.

Abbreviations: CI, confidence interval; FOS, fish oil supplements; HR, hazard ratio; P, *p*-values for association between interaction loci and dementia outcomes.

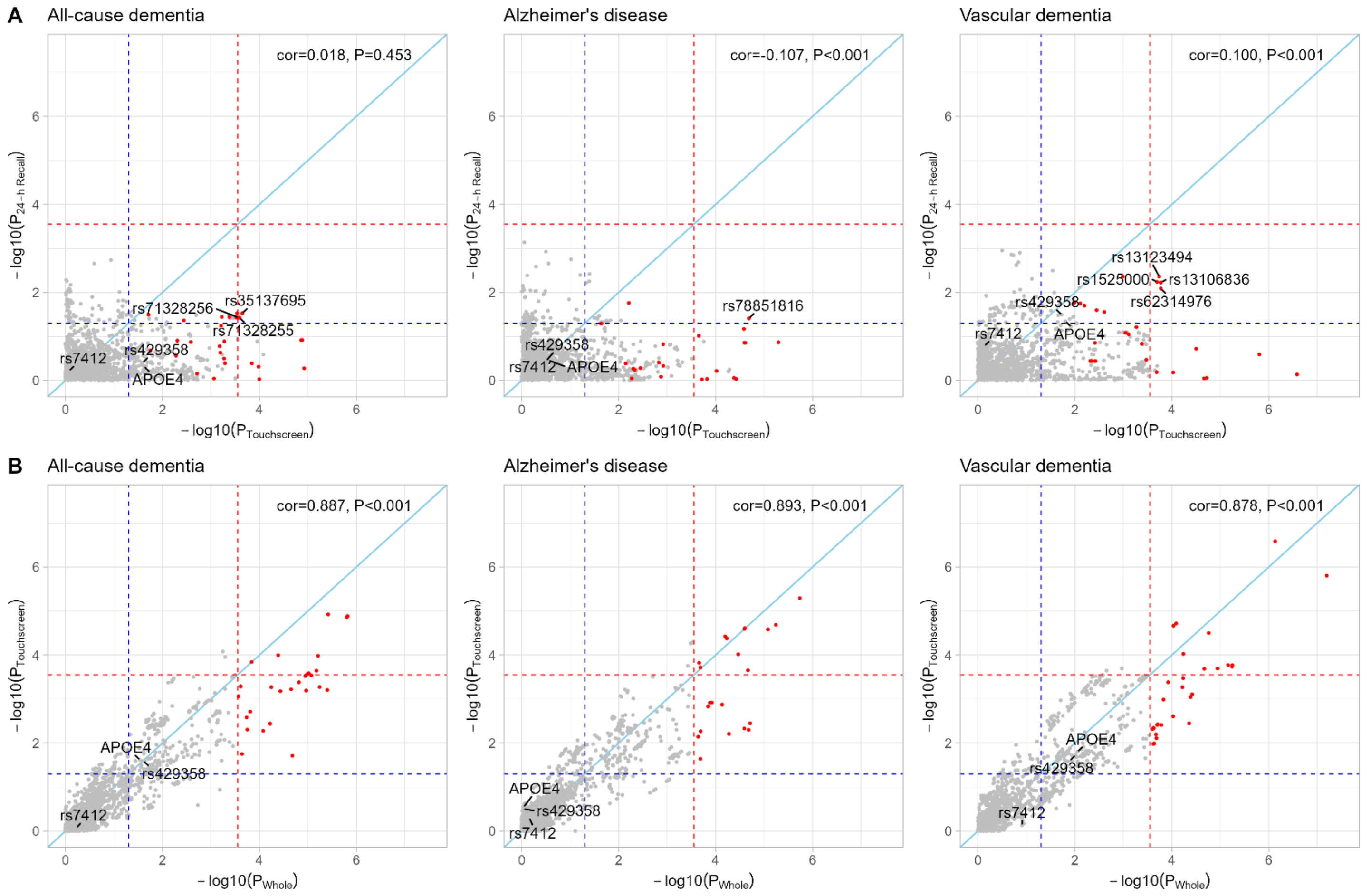

Figure S7. Interaction analysis of candidate SNPs with FOS status from the touchscreen questionnaire and 24-hour recall questionnaire (model 1).

A) Comparison of p-values for SNP × FOS interaction inferred from exclusive touchscreen questionnaire (N = 201,627) and 24-hour recall questionnaire (N = 156,004). B) Comparison of p-values for SNP × FOS interaction between the whole dataset (N = 357,631) and exclusive touchscreen questionnaire (N=201,627). SNPs that significantly interacted with FOS status in the whole dataset were demonstrated by red points. The Red dashed line and blue dashed line referred to the Bonferroni correction threshold ($p = 2.8 \times 10^{-4}$) and nominal significance threshold ($p = 0.05$), respectively. Pearson tests were conducted to assess the correlation of *p*-values between the two datasets.

Abbreviations: cor, Pearson correlation coefficient; P, *p*-value of Pearson correlation test; P_{whole}, $P_{touchscreen}$, and $P_{24\text{-h Recall}}$, *p*-values of the interaction terms.

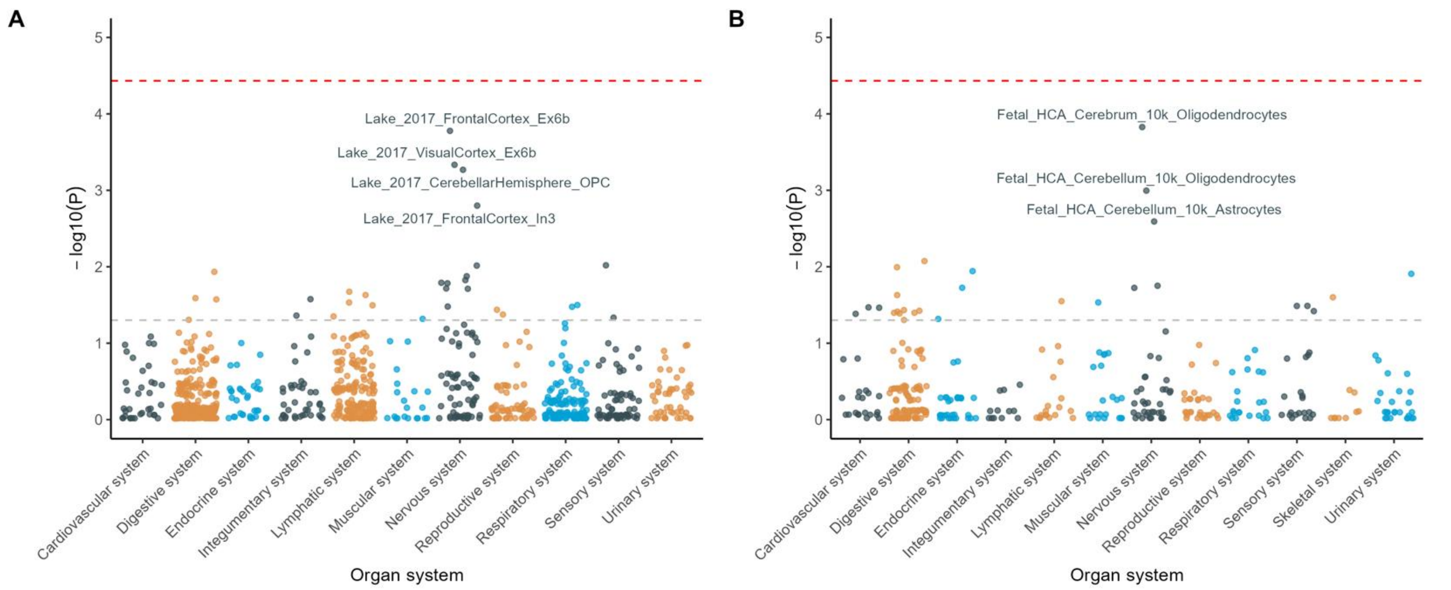

Figure S8. Gene-set enrichment analysis of cell-type specific expression for candidate interacting, protein-coding genes, stratified by adult and fetal tissues.

A) Enrichment signals across 941 adult tissue-cell types. B) Enrichment signals across 364 fetal tissue-cell types. Red and grey dashed lines represent thresholds for Bonferroni-corrected significance (0.05/total number of tissue-cell types) and nominal significance (0.05), respectively.

Abbreviations: Ex6b, layer 6b excitatory neuron subtype; HCA, human cell atlas; In3, layer 3 inhibitory neuron subtype; OPC, oligodendrocyte progenitor cell; P, permutated p-value of gene-set enrichment analysis.

Reference

[1] Bellenguez C, Kucukali F, Jansen IE, Kleineidam L, Moreno-Grau S, Amin N, et al. New insights into the genetic etiology of Alzheimer's disease and related dementias. Nat Genet. 2022;54(4):412-36. doi: 10.1038/s41588-022-01024-z.

[2] Karjalainen MK, Karthikeyan S, Oliver-Williams C, Sliz E, Allara E, Fung WT, et al. Genome-wide characterization of circulating metabolic biomarkers. Nature. 2024;628(8006):130-8. doi: 10.1038/s41586-024-07148-y.

[3] Richardson TG, Leyden GM, Wang Q, Bell JA, Elsworth B, Davey Smith G, Holmes MV. Characterising metabolomic signatures of lipid-modifying therapies through drug target mendelian randomisation. PLoS Biol. 2022;20(2):e3001547. doi: 10.1371/journal.pbio.3001547.

[4] Francis M, Sun Y, Xu H, Brenna JT, Ye K. Fifty-one novel and replicated GWAS loci for polyunsaturated and monounsaturated fatty acids in 124,024 Europeans. medRxiv. 2022:2022.05.27.22275343. doi: 10.1101/2022.05.27.22275343.

[5] Borges MC, Haycock PC, Zheng J, Hemani G, Holmes MV, Davey Smith G, Hingorani AD, Lawlor DA. Role of circulating polyunsaturated fatty acids on cardiovascular diseases risk: analysis using Mendelian randomization and fatty acid genetic association data from over 114,000 UK Biobank participants. BMC Med. 2022;20(1):210. doi: 10.1186/s12916-022-02399-w.

[6] Davyson E, Shen X, Gadd DA, Bernabeu E, Hillary RF, McCartney DL, Adams M, Marioni R, McIntosh AM. Metabolomic Investigation of Major Depressive Disorder Identifies a Potentially Causal Association With Polyunsaturated Fatty Acids. Biol Psychiatry. 2023;94(8):630-9. doi: 10.1016/j.biopsych.2023.01.027.
